# Construction of a novel reporter strain indicating inhibition of cell wall biosynthesis

**DOI:** 10.1007/s00253-026-14013-7

**Published:** 2026-08-28

**Authors:** Maike Laura Karcher, Annette Siskowski, Chantal Müller, Laura Engel, Vincent Franz, Susanne H. Kirsch, Birthe Sandargo, Tanja Schneider, Gabriele Bierbaum

**Affiliations:** 1 Institute of Medical Microbiology, Immunology and Parasitology, University Hospital Bonn, Venusberg-Campus 1, Bonn, 53127 Germany; 2 Institute for Pharmaceutical Microbiology, University Hospital Bonn, University of Bonn, 53115 Bonn, Germany; 3 Helmholtz Centre for Infection Research (HZI), Helmholtz Institute for Pharmaceutical Research Saarland (HIPS), 66123 Saarbrücken, Germany; 4 German Centre for Infection Research (DZIF), Partner Site Hannover-Braunschweig, 38124 Braunschweig, Germany; 5 Helmholtz Centre for Infection Research (HZI), 38124 Braunschweig, Germany; 6 German Center for Infection Research (DZIF), Partner Site Bonn-Cologne, 53115 Bonn, Germany

**Keywords:** Luciferase reporter assay, WalRK (YycFG) two-component system, LiaRS stress response, Cell envelope stress, PrkC kinase, Cell wall biosynthesis

## Abstract

**Abstract:**

The WalRK two-component system monitors cell wall biosynthesis, and its activity decreases when biosynthesis is inhibited. As WalRK is essential for many Gram-positive bacteria, including *Staphylococcus aureus* and *Bacillus subtilis*, directly inhibiting WalK would disrupt cell wall biosynthesis and result in cell death. This study presents the construction of new reporter strains with two WalRK-dependent promoters: P*iseA* and P*ssaA*. These reporter strains enable indirect in vivo measurement of WalRK activity based on luminescence. We evaluated these reporters in combination with the established P*liaI* reporter system, which is regulated by LiaRS, in order to improve the identification of cell wall inhibitors during the screening of natural products. The WalRK-dependent P*iseA* reporter yielded specific responses to almost all known cell wall biosynthesis inhibitors, including β-lactams, tunicamycin, glycopeptides, daptomycin, and fosfomycin, with no discernible response to other antibiotic classes. Furthermore, combining the P*liaI* reporter system with the P*iseA* reporter enhanced the range of potential cell wall inhibitors that could be detected. Deleting the gene of the eukaryotic-like Ser/Thr kinase PrkC increased the P*iseA* signal obtained with certain antibiotics, as well as after a longer incubation period. Additionally, deletion of *prkC* lowered P*liaI* expression in the presence of lipid II-targeting antibiotics. Screening natural products with an unknown mode of action identified several compounds that elicited vancomycin-like patterns with the reporter systems, indicating potential cell wall inhibitory activity. Overall, combining WalRK and LiaRS reporters enables a rapid, complementary evaluation of cell wall stress responses, providing a powerful method for discovering new cell wall-targeting antibiotics.

**Key points:**

• *The PiseA/PssaA reporters enable continuous in vivo observation of WalRK activity*

• *Cell envelope stress responses are differentially sensed by WalRK and LiaRS*

• *The PiseA reporter strain detects nearly all cell wall biosynthesis inhibitors*

**Supplementary Information:**

The online version contains supplementary material available at https://doi.org/10.1007/s00253-026-14013-7.

## Introduction

*Staphylococcus aureus* is a common opportunistic pathogen which causes life-threatening nosocomial infections (Reddy et al. 2017). In 2019, *S. aureus* caused an estimated 1.1 (0.8–1.4) million deaths worldwide and, therefore, reaches the level of *M. tuberculosis* (1.2 million deaths) (Ikuta et al. 2022). Methicillin-resistant *S. aureus* (MRSA) is still a major health care issue and are listed as a high-priority pathogen by the WHO (World Health Organization 2024). Consequently, the discovery of new antibiotics is important. To this end, model organisms that are conducive to genetic analysis are frequently utilised to develop reporter systems that facilitate the effective identification of novel antibacterial agents and their mode of action (e.g. Mascher et al. 2003; Kobras et al. 2017). *B. subtilis* represents a well-established Gram-positive model organism that is non-pathogenic and naturally competent (Spizizen 1958) and has served as model for many sophisticated studies on antibiotic mode of action (Hasper et al. 2006; Müller et al. 2016). Furthermore, *B. subtilis* shares highly conserved cell envelope stress response pathways with clinically relevant pathogens, including *S. aureus* (Kuroda et al. 2003; Jordan et al. 2008).

Bacteria perceive environmental conditions and cellular status using two-component systems (TCS). TCS function as chemo-, osmo- and temperature-sensors, and adapt cellular responses to stress conditions by up- and/or downregulation of their regulons (Fabret and Hoch 1998; Hoch 2000). A basic TCS comprises a histidine kinase, typically with an extracellular sensing domain, and a response regulator, which is activated by phosphorylation by the kinase. Often additional proteins are involved, like WalH and WalI in the WalRK-TCS (Szurmant et al. 2007). In antibiotic discovery, monitoring the activities of TCS in a bacterial cell will convey an exact picture of the stresses that are mediated by antibiotics and, therefore, indicate the mode of action of a new antibiotic compound.

The TCS LiaRS, which is functionally related to the VraRS cell envelope stress response system in *S. aureus* (Kuroda et al. 2003), has been successfully utilised in screening for cell wall inhibitors that affect the lipid II cycle and membrane integrity (Mascher et al. 2004). Especially in the presence of antibiotics like vancomycin and bacitracin, LiaRS induces the promoter of the *liaIHGFSR* operon, causing a cell envelope-associated stress response (Mascher et al. 2004; Jordan et al. 2006). However, β-lactams only elicit a very moderate response of the P*liaI* reporter strains (Mascher et al. 2004). In contrast, the reporter strains of Lautenschläger et al. (2020) constitute specific β-lactam biosensors in *Bacillus subtilis* using the heterologous regulatory system BlaR1I from *S. aureus*. Additionally, the use of the σ^M^-mediated regulatory system (Yin et al. 2022) and the *ypuA* promoter-reporter fusion (Urban et al. 2007) provides a simplified, rapid method for identifying potential cell wall inhibitors via specific reporter systems. In order to complement existing reporter systems and expand the detection of cell wall biosynthesis inhibitors, a reporter-based system using the regulatory system WalRK could provide an additional perspective on cell wall homeostasis and inhibition of peptidoglycan biosynthesis.

WalRK (formerly YycFG or VicRK) is the only essential TCS in Gram-positive bacteria with low G + C content like *S. aureus*, *Enterococcus faecalis*, *Listeria monocytogenes*, *Streptococcus pneumoniae*, and *Streptococcus mutans* and was originally described in *B. subtilis* (Fabret and Hoch 1998; Bisicchia et al. 2007; Dubrac et al. 2007, 2008). In *B. subtilis*, WalK detects cleavage products generated by the endopeptidases CwlO and LytE (Dobihal et al. 2019). WalR is the cognate response regulator of WalK that binds directly to promoter regions with the binding motif 5´TGT(A/T)A(A/T/C)-N_5_-TGT(A/T)A(A/T/C)-3´ in *B. subtilis*, and activates or in rare cases inhibits transcription of WalRK-regulated genes (Bisicchia et al. 2007). The orthologues of WalR in Gram-positive bacteria with low G + C content, e.g., *S. aureus*, display a high level of amino acid sequence identity (generally > 70%), especially in the DNA-binding domain (Howell et al. 2003; Dubrac and Msadek 2004; Dubrac et al. 2008; Sharkey et al. 2023). Due to protein/DNA interaction studies in Dubrac and Msadek (2004), the same binding motif was also identified in *S. aureus*. The numbers of additional proteins that are included in the sensory complex differ between species (Wagner et al. 2002; Szurmant et al. 2007).

WalRK is essential for bacterial growth and survival with a central role in cell envelope homeostasis involving the regulation of cell wall metabolism as well as activating the expression of the majority of *S. aureus* autolysins (e.g., LytM, SleI, Atl, SceD, SsaA, IsaA) (Dubrac and Msadek 2004; Dubrac et al. 2007). In *B. subtilis*, WalRK also activates the expression of autolysin genes such as *yocH*, *cwlO*, *lytE*, *ykvT* and *ydjM* (Howell et al. 2003; Szurmant et al. 2005; Bisicchia et al. 2007). The gene *iseA* encodes an inhibitor of autolysin activity, targeting the D,L-endopeptidases LytE, LytF and CwlS and is negatively regulated by WalRK in *B. subtilis*, since WalR binds within the core promoter region overlapping the -35 and -10 element, thereby inhibiting transcription (Bisicchia et al. 2007; Dubrac et al. 2007, 2008; Salzberg and Helmann 2007; Dobihal et al. 2022). Additionally, the WalRK regulon is influenced by the eukaryotic-like Ser/Thr kinase PrkC, which phosphorylates WalR at Thr101 in vivo and in vitro (Libby et al. 2015). The PASTA-containing kinase PrkC is involved in sensing cell envelope-associated signals, including cell wall-derived muropeptides and its activation mechanism seems to be growth-phase dependent in *B. subtilis* (Squeglia et al. 2011; Pompeo et al. 2018). Indeed, WalR is phosphorylated simultaneously by WalK at Asp53 and PrkC at Thr101. The secondary phosphorylation at Thr101 increases both the activation and repression of WalR regulon genes (Libby et al. 2015). During the transition into stationary phase, the effect of PrkC on the expression of the WalR regulon is most significant, because WalK activity decreases in this growth phase (Fabret and Hoch 1998). Direct in vivo monitoring of WalRK activity remains challenging, particularly in screening approaches, given that WalRK is a key regulator of cell wall homeostasis and that—in contrast to other two-component systems—its transcription is not autoregulated (Fabret and Hoch 1998). Consequently, the transcriptional responses of WalRK-regulated genes must provide a readout of WalRK activity. For this reason, the promoters of *iseA* and *ssaA*, which are negatively and positively regulated by WalRK respectively, were chosen as suitable WalRK-associated targets (Dubrac and Msadek 2004; Bisicchia et al. 2007; Salzberg and Helmann 2007). An additional advantage of using the genetically tractable model organism *B. subtilis* is that—in contrast to *S. aureus*—there are two genes (*iseA* and *pdaC*) whose transcription is inhibited by phosphorylated WalR (Bisicchia et al. 2007), so that reporter strains with these promoters will enable a positive read-out of a decrease of WalK activity.

For further identification of new inhibitors of cell wall biosynthesis, we describe the construction of reporter strains harbouring WalRK-dependent promoters fused to the *luxCDABE* operon of *Photorhabdus luminescens*, to monitor effects on cell wall biosynthesis sensed by WalRK activity in vivo and enable continuous monitoring of WalRK activity. The reporter strains were validated using antibiotics with different modes of action. In addition, new myxobacterial and fungal antibiotics were tested in vitro and in vivo for their effects on WalRK in comparison to the P*liaI* reporter strain from Kobras et al. (2017). Additionally, the PrkC-dependent regulation of WalRK regulated genes was investigated after deleting the *prkC* gene within the reporter strains. The aim of this study was to develop and validate WalRK-dependent reporter strains that complement the P*liaI* system using the same experimental basis, enabling the detection of direct WalK inhibition and facilitating the identification of novel cell wall-targeting antibiotics.

## Materials and methods

### Antibacterial compounds

The assays included 66 myxobacterial and fungal natural products, a subset of the DZIF Natural Product Library located at the Helmholtz-Institute for Pharmaceutical Research Saarland (HIPS) (Supplemental Table S7).

### Cloning of the promoter-*luxCDABE* fusions in *B. subtilis* 168

Four promoter regions, including their upstream regions, of WalRK regulated genes were fused to the *lux*-operon of *Photorhabdus luminescens* in *B. subtilis* 168. The promoter regions of *iseA*, *ykvT*, and *pdaC* were amplified from the genome of *B. subtilis* 168, using the primers iseA_for_NotI, iseA_rev_SalI, ykvT_for_NotI, ykvT_rev_SalI, pdaC_for_NotI, and pdaC_rev_SalI, and *ssaA* was obtained from *S. aureus* SG511, respectively, using the primers ssaA_for_NotI, and ssaA_rev_SalI listed in Supplemental Table S1. The plasmid pCHlux101-P*liaI* (Kobras et al. 2017), harbouring the *luxCDABE* operon, was digested with the restriction enzymes *Not*I/*Sal*I resulting in deletion of the promoter region of *liaI*. The promoter regions were then amplified by PCR, digested with *Not*I/*Sal*I and ligated into pCHlux101. Subsequently, the newly created plasmids were linearised and transformed into competent *B. subtilis* 168 (Radeck et al. 2013). The plasmids contained fragments of *sacA* on both sides of the luminescence gene cluster and were integrated into *sacA* by double homologous recombination. This strategy positions all reporter constructs at the same chromosomal location as the established P*liaI* reporter, ensuring stable single-copy expression in identical genetic surroundings and allows direct comparison of the different reporters. Integration at the native loci was avoided to prevent disruption of the physiological regulation of the endogenous WalRK target genes (Radeck et al. 2013; Kobras et al. 2017). Integration was confirmed by PCR with the primers pBs3Clux-for and pBs3Clux-rev and whole genome sequencing. Primer sequences and bacterial strains are listed in Supplemental Table S1 and Supplemental Table S2, respectively. Whole genome sequencing was performed by GENEWIZ®, Leipzig, Germany. The DNA for whole genome sequencing was purified with the FastDNA™ SPIN Kit for Soil from MP Biomedicals, according to the instructions.

### Luminescence assay

Expression of different WalK-dependent promoters was determined by luciferase activity. Additionally, *B. subtilis* 168 pCH*lux*101-P*liaI* (Kobras et al. 2017) was used as a control to detect cell wall stress-inducing compounds. Overnight cultures were diluted 1:100 in MH medium with 5 µg/mL chloramphenicol and grown at 30 °C with shaking to an OD_600_ of 0.5–1. For measurement of mersacidin activity, 2 mM Ca^2+^ was added, whereas a final concentration of 0.5 mM Ca^2+^ was used for daptomycin. Separate control cultures in the absence of antibiotics were performed for every antibiotic treatment. Serial twofold dilutions of the antibiotics with a volume of 50 µl were inoculated with 50 µL suspensions of the reporter strains (4 × 10^8^ to 1 × 10^9^ cells per mL) in white 96-well clear-flat-bottom polystyrene microtiter plates (Greiner Bio-One, Frickenhausen, Germany). Previously, 50 µL MH medium had been added into the plate. The cells were incubated at 30 °C for 3 h, measuring the optical density and the luminescence every 10 min. For evaluation of the results, the luminescence in presence and absence of the compounds was divided by the optical density in the wells to yield luminescence per cell. To evaluate the induction of luminescence by the compounds, the luminescence/cell obtained with the reporter strains was divided by the luminescence/cell of the control grown in absence of the antibiotics and expressed in percent signal induction. All experiments were performed in triplicate, with two technical replicates for each biological replicate. Mean values and standard deviation (SD) were calculated using Excel. Welch's t-test was used to compare signal induction employing RLU/OD values of the treated culture compared to the RLU/OD of the individual growth control, or the Δ*prkC* mutant versus the parent strain. The results are presented as follows: P < 0.001; ***P < 0.01; **P < 0.05; *. The MIC values were determined for both the wild-type and the Δ*prkC* reporter strains. As no differences in MIC values were observed between the strains, the same antibiotic concentrations were used for the corresponding conditions in subsequent reporter assays (Supplemental table S3).

### Knockout of *prkC* in the *B. subtilis* reporter strains

To test the influence of PrkC on the reporter strains, the *prkC* gene was deleted. A fragment (2.250 bp) was amplified by PCR, consisting of a left arm (1,650,845 to 1,651,344 in NC_000964), an erythromycin resistance gene (*ermB*) and a right arm (1,652,887 to 1,653,386 in NC_000964). The corresponding primers are listed in Supplemental Table S1. For the amplification of the *ermB* gene, the plasmid pMUTIN4 was utilised. The PCR fragment was integrated into the *B. subtilis* reporter strains P*liaI* and P*iseA* via competence transformation and the correct insertion was verified via sequencing. Additionally, the *prkC* deletion mutants and the original reporter strains were characterised by whole genome sequencing as described above.

### Antimicrobial susceptibility testing

The minimal inhibitory concentration (MIC) of the different antibiotics was performed in polystyrene round-bottom microtiter plates (Greiner Bio-One, Frickenhausen, Germany). The cells were incubated in Müller-Hinton (MH) broth with an inoculum of 5 × 10^5^ CFU/ml and utilised for the broth microdilution method. The MIC was defined as the lowest concentration of antibiotics that inhibited visible growth after 24 h and 48 h incubation at 30 °C.

### Cloning, overexpression and purification of *S. aureus* WalK and WalR

Overexpression and purification of *S. aureus* WalK and WalR were performed as described in Türck and Bierbaum (2012).

### In vitro phosphorylation assay with Phos-tag acrylamide

The phosphorylation assays of WalK and the Phos-tag PAGE were performed as described previously (Dietrich et al. 2023). In short, the assays were performed with a sample volume of 12 µl to test a potential inhibiting compound at different concentrations. The ingredients in each reaction tube were 0.5 µg of the histidine kinase, 1 µg of the corresponding response regulator, 1.2 µl 85 mM Triton X-100 (final concentration 8.5 mM), dialysis buffer (dependent on kinase concentration), 2.4 µl 5 × phosphorylation buffer (250 mM HEPES, 2.5 M KCl, 25 mM MgCl_2_, 17.5% glycerol, 2.5 mM DTT added shortly before use, pH 8) and ultrapure water to yield a final volume of 12 µl. Different dilutions of the compounds to be tested were prepared, and 1 µl was added to each reaction. Some of the compounds were dissolved in DMSO; therefore, a solvent control, containing 8% DMSO which corresponds to the highest concentration in the assay, was performed and did not show any effect. The assay was incubated for 10 min at 15 °C, before the reaction was started by addition of 5 mM ATP. The reaction was incubated for 30 min, samples were taken after 0 min, 10 min and 30 min. Additionally, a negative control without ATP was performed. To stop the reaction, 12 µl of 2 × Laemmli SDS sample buffer diluted with 1:20 β-mercaptoethanol was mixed with 12 µl of the assay.

The Phos-tag gel was prepared like a normal SDS-PAGE except that 5 mM Phos-tag acrylamide (FUJIFILM Wako Pure Chemical Corporation, Richmond, VA, USA, Cat. #304‑93,521) and 10 mM MnCl_2_ were added to the 12.5% resolving gel. The gel was stained with Coomassie Brilliant Blue R250 (Methanol 50% (v/v), Acetic acid 10% (v/v), Coomassie brilliant blue 0.25% (w/v)) for 30 min and decolourisedd with destaining solution (Methanol 20% (v/v), Acetic acid 10% (v/v)), which was replaced regularly.

## Results

### The activity of the WalK reporters varies during growth

**Fig. 1:**
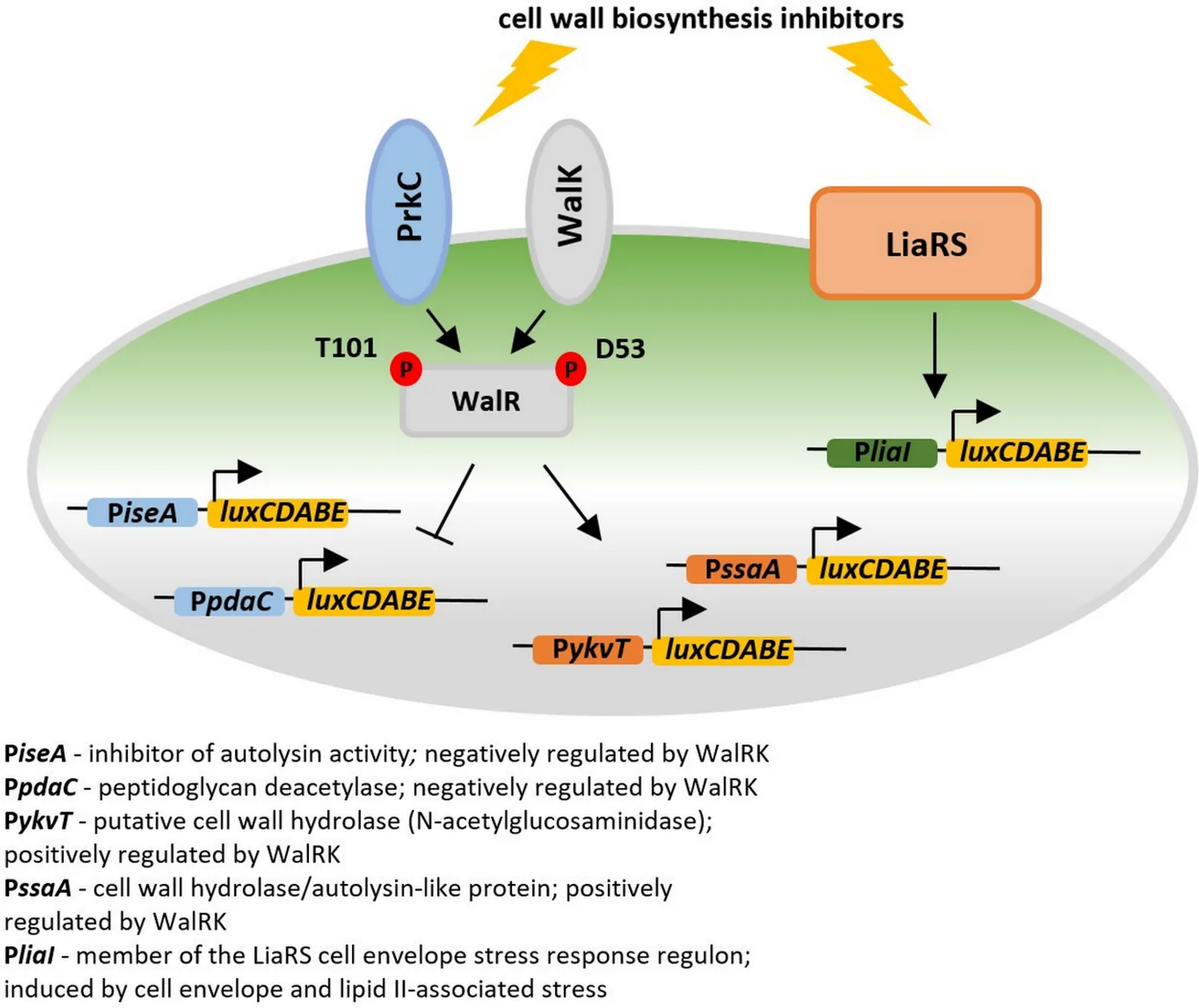
Schematic overview of the regulatory relationships between WalRK, PrkC, and the reporter promoters used in this study. WalRK represses the transcription of P*iseA*/P*pdaC* and activates P*ssaA*/P*ykvT *expression. PrkC enhances WalR activity through phosphorylation. The LiaRS system activates P*liaI* in response to cell envelope stress by antibiotics, whereas WalRK responds to antibiotic interference with cell wall biosynthesis (flash arrows). Black arrows indicate positive regulation and blunt-ended arrows indicate negative regulation

**Fig. 2:**
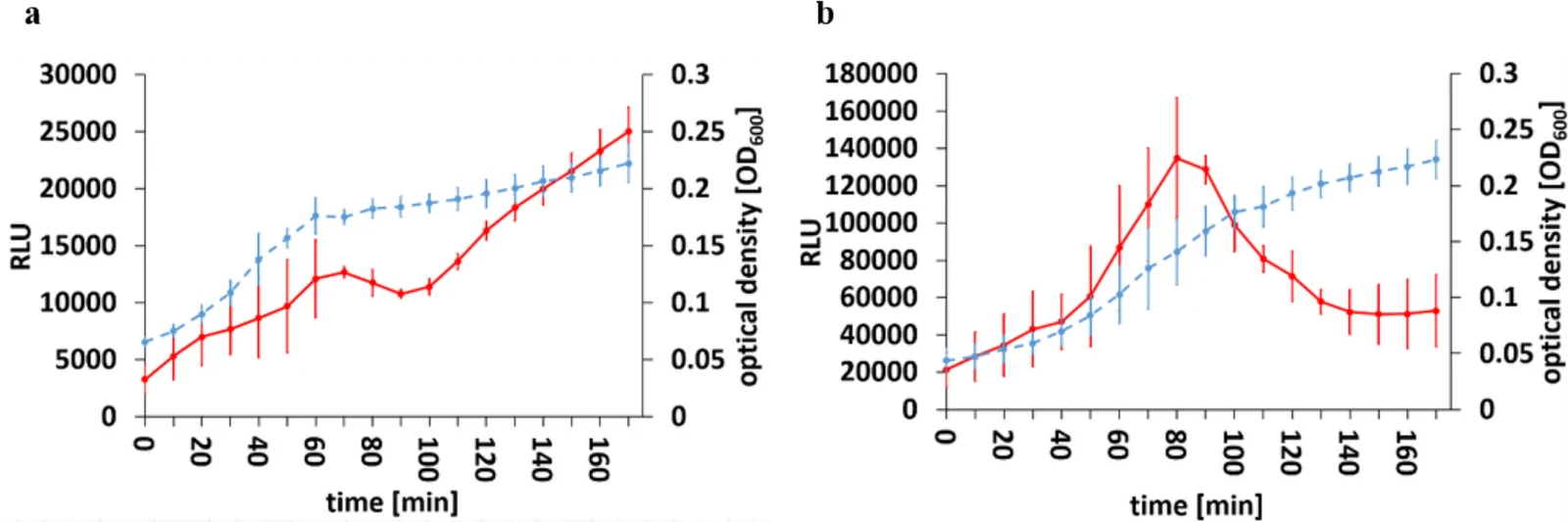
Luminescence signal (RLU) over time and associated growth curves of WalRK-dependent reporter strains: P*iseA* (**a**) and P*ssaA* (**b**) in *B. subtilis* 168, representing WalRK activity. *B. subtilis* pCHlux101-P*iseA*, and *B. subtilis* pCHlux101-P*ssaA* were grown to exponential phase and added to a 96-well plate. The assay was measured in a TECAN plate reader for 3 h, measuring OD_600_ (blue) and luminescence (RLU) every 10 min (red)

The promoter-*luxCDABE* fusions in *B. subtilis* 168 were created to observe the activity of the WalRK system in the presence of different antibiotics and identify cell wall biosynthesis and kinase inhibitors. The regulatory relationships underlying these reporter systems, including the influence of PrkC and LiaRS, are summarised in Fig. 1. The promoter regions of *iseA* and *pdaC* from *B. subtilis* were selected because these are the only genes whose transcription is inhibited by WalRK (Bisicchia et al. 2007), so that, in turn, an increase of expression of P*iseA* and P*pdaC* indicates a lower activity of the TCS. The positively regulated promoter regions of *ssaA* from *S. aureus* and *ykvT* from *B. subtilis* were selected to show a possible activation of the WalRK system in the presence of antibiotics as described in Cheng et al. (2024). WalRK is always active in the exponential growth phase and downregulated in the stationary phase (Fig. 2). Consequently, the luminescence of the P*iseA* reporter, increased disproportionately after 100 min (Fig. 2a), when the cells entered stationary phase (Fig. 2a). The expression of P*ssaA* was, compared to P*iseA*, highest in exponential phase and reached its maximum after 80 min, indicating that the consensus promoter sequence of *S. aureus* was recognised by the WalR of *B. subtilis* (Fig. 2b).

### The P*iseA*-reporter is activated by glycopeptides, β-lactams, tunicamycin, daptomycin and fosfomycin

In following experiments, the expression of the WalK reporters was compared to a third reporter strain with the *liaI* promoter, that indicates inhibition of cell wall biosynthesis by compounds targeting the cell wall precursor lipid II as well as compounds that lead to membrane perturbations (Cao et al. 2002; Mascher et al. 2003, 2004; Hutter et al. 2004).

**Fig. 3:**
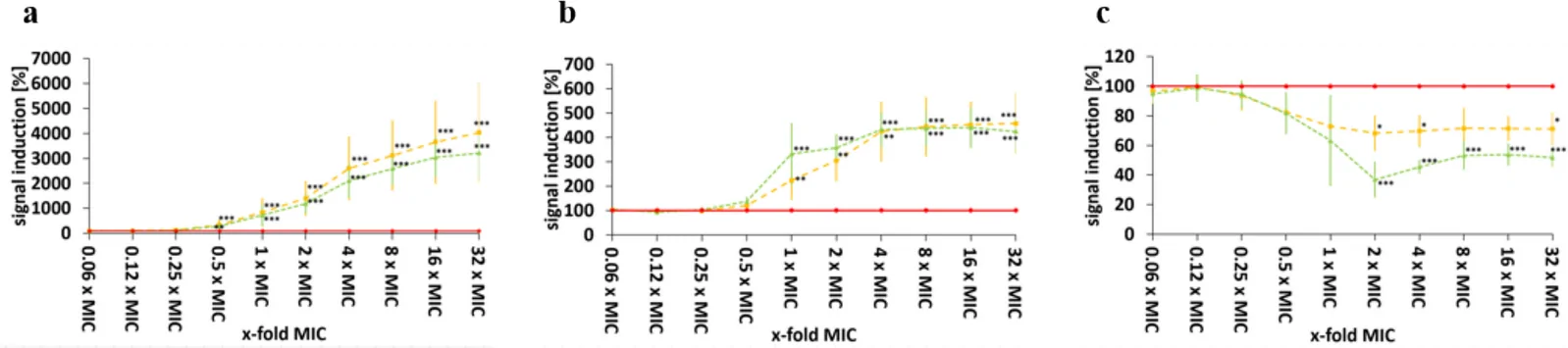
Expression levels of P*liaI* (**a**), P*iseA* (**b**) and P*ssaA* (**c**) represented as signal induction in percent (%) against the vancomycin concentration (fold MIC) and normalised to growth control (red). *B. subtilis* pCHlux101-P*liaI*, *B. subtilis* pCHlux101-P*iseA*, *B. subtilis* pCHlux101-P*ssaA* were grown to exponential phase and added to a 96-well plate with different x-fold MIC concentrations of vancomycin. The assay was measured in TECAN plate reader for 3 h, measuring OD_600_ and luminescence (RLU) every 10 min. Values obtained after 30 (yellow) and 60 (green) minutes are shown. Welch´s t-test was performed to compare the data sets to the growth control. P < 0.001; ***P < 0.01; **P < 0.05; *

Since WalK is an essential system (Fabret and Hoch 1998), a signal is also generated in the absence of an antibiotic, as shown in Fig. 2. Therefore, the data in Fig. 3 and subsequent figures show signal induction for all three strains in percent (%), normalized to the growth control, against different antibiotic concentrations (expressed as fold MIC, Supplemental Table S3). In total, 200% of expression was set as a tentative threshold for induction of the reporters; values of 100% were consistent with expression of the growth control and signals under 100% mean downregulation of the expression.

As expected, vancomycin induced an extremely high expression up to 4000% of P*liaI* after 30 min and 3200% after 60 min at 32 × MIC/ 8 µg/ml (Fig. 3a). The expression of P*iseA* was also increased up to 450% after 30 min and 420% after 60 min at the maximum concentration. (Fig. 3b), indicating a downregulation of the WalRK system (Supplemental Figure S2). In addition, vancomycin decreased P*ssaA* expression (Fig. 3c) as predicted, confirming that the P*iseA* and P*ssaA* reporter strains showed contrasting expression patterns. In contrast, the fold-induction signals obtained with the promoter sequences of *pdaC* and *ykvT* in the presence of vancomycin were considerably lower and showed no strong reaction in the presence of vancomycin (Supplemental Figure S3). In the case of P*pdaC*, this may be due to the unexpectedly high basal expression in the control compared to the other promoters (Supplemental Figure S1; RLU measured after 60 min in the control culture: P*liaI*, 656; P*iseA,* 9704; P*pdaC,* > 50.000). As we wanted to measure upregulation of transcription in the presence of antibiotics in comparison to the control culture (‘fold induction’) and because the production of the encoded enzymes producing luminescence might also constitute a fitness cost that influences the measurements, we chose P*iseA* for further experiments.

In contrast, transcription of P*ykvT* was very low (Supplemental Figure S3), although presenting the predicted growth phase dependent pattern (Howell et al. 2003) and no downregulation in the presence of vancomycin was observed. Since *ssaA* of *S. aureus* represents a well-characterised WalRK-activated autolysin-associated gene (Dubrac and Msadek 2004) and a significant effect of vancomycin and the predicted growth-phase dependent expression pattern in the control were observed in *B. subtilis*, the *ssaA* promoter was selected as an alternative WalRK-activated reporter. Consequently, the reporter strains with P*iseA* and P*ssaA* are presented in the subsequent experiments.

In contrast to WalK, LiaS is not active during exponential growth and only activated in the presence of compounds inducing *liaI* promoter. Although the luminescence of the P*iseA* reporter, measured as relative light units (RLU), was always higher than that of the P*liaI* reporter, its strong basal activity led to fold-induction values that were always lower than those of P*liaI* (Supplemental Table S4).

**Fig. 4:**
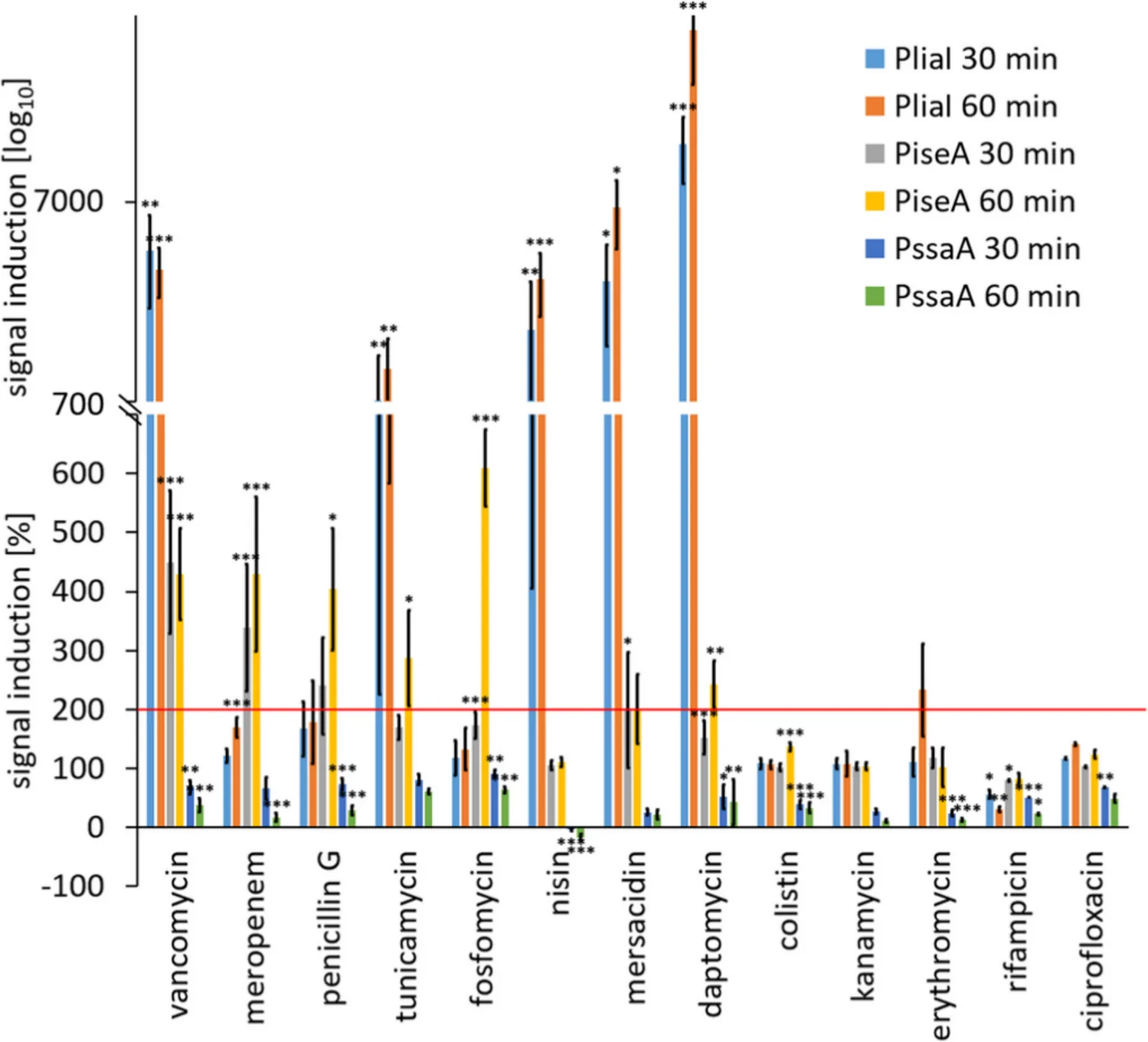
Maximal upregulation of P*liaI*, P*iseA* and downregulation of P*ssaA* in the presence of various antibiotics. *B. subtilis* pCHlux101-P*liaI*, *B. subtilis* pCHlux101-P*iseA*, *B. subtilis* pCHlux101-P*ssaA* were grown to exponential phase and added to a 96-well plate with different x-fold MIC concentrations of the antibiotics. The assay was measured in TECAN plate reader for 3 h, measuring OD_600_ and luminescence (RLU) every 10 min. Mean values and their standard deviation (SD) obtained after 30 and 60 min are shown. The experimental cut-off is 200%. The higher values are presented in log_10_. Welch´s t-test was performed to compare the data sets to the growth control. P < 0.001; *** P < 0.01; ** P < 0.05; *

Figure 4 shows the maximal upregulation of P*liaI* and P*iseA*, as well as the maximal downregulation of P*ssaA* in presence of well-established antibiotics; Supplemental Table S5 lists the antibiotic concentrations with the highest efficacy in this test. Cell wall biosynthesis inhibitors (β-lactams, glycopeptides, fosfomycin and tunicamycin) induced an upregulation of the P*iseA* promoter after 60 min, when the cells were in the late exponential phase (Fig. 2). The most effective vancomycin concentrations in this experiment were higher than the MIC. This is caused by the distinctive inoculum effect of vancomycin, since the inoculum was more than 1,000-fold higher than in a MIC determination (4 × 10^8^ to 1 × 10^9^ cells per ml) (Felmingham et al. 1987; LaPlante and Rybak 2004). In contrast, P*liaI* was not induced by β-lactams and fosfomycin.

The lantibiotics nisin and mersacidin also target lipid II (Brötz et al. 1998; Breukink and Kruijff 1999). Mersacidin induced strong P*liaI* expression and just reached the 200% threshold for P*iseA*. In addition to binding to lipid II, nisin also forms pores in the cytoplasmic membrane (Wiedemann et al. 2001) and sequestrates lipid II into raft-like membrane domains (Hasper et al. 2006). Both processes lead to membrane damage, which causes a general inhibition of cellular biosynthetic processes (Wiedemann et al. 2001; Hasper et al. 2006; Tol et al. 2015). Nisin strongly induced the P*liaI* reporter; however, the activity of WalRK-dependent reporter P*iseA* remained below the 200% threshold, although significantly upregulated. Daptomycin forms Ca^2+^-dependent complexes with phosphatidylglycerol and prenyl-coupled cell wall precursors, including lipid II, and causes dissociation of MurG from the cytoplasmic membrane of *B. subtilis*, thereby it inhibits cell wall biosynthesis and leads to a simultaneous loss of membrane potential (Müller et al. 2016; Grein et al. 2020). It elicited a modest response of P*iseA* and a strong response of P*liaI* as already described by Wecke et al. (2009).

Colistin, a polymyxin antibiotic, that attacks the cytoplasmic membrane in *Bacillus* (Yu et al. 2015), did not stimulate the P*iseA* and P*liaI* reporters. The same results were obtained for inhibitors of protein, RNA, and DNA biosynthesis (Supplemental Figure S4). In contrast, P*ssaA* expression was predominantly downregulated in presence of almost all antibiotics, suggesting that a decreased signal of the P*ssaA* reporter is unspecific for cell wall stress and may reflect reduced cellular activity associated with growth inhibition or antibiotic-induced stress (Fig. 4, Supplemental Table S5).

### PrkC affects signal strength of P*iseA* and P*liaI* in the presence of vancomycin

**Fig. 5:**
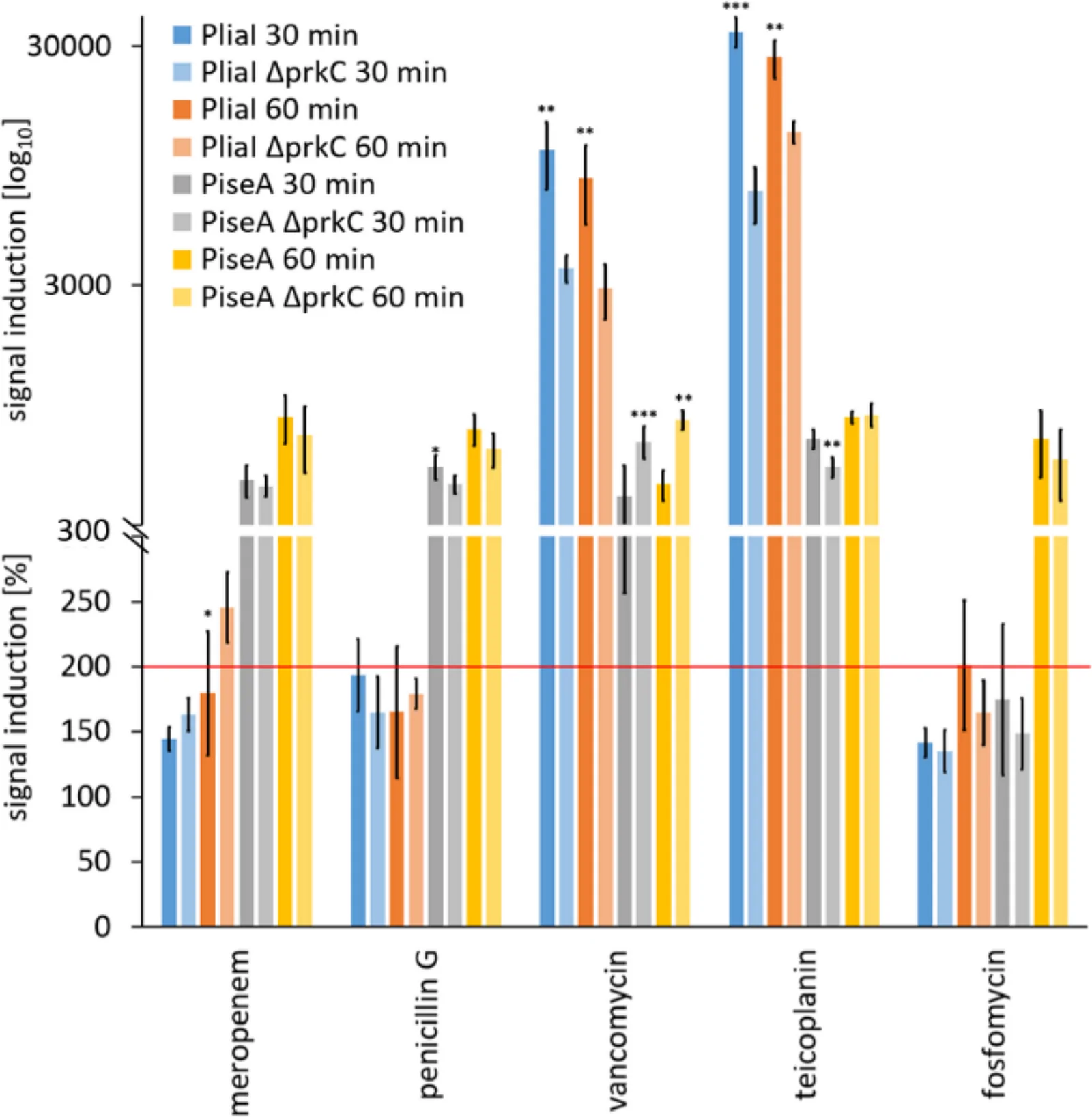
Maximal upregulation of P*liaI* and P*iseA,* compared to *prkC* mutants in the presence of various antibiotics, after 30 and 60 min. *B. subtilis* pCHlux101-P*liaI*, *B. subtilis* pCHlux101-P*liaI*∆*prkC*, *B. subtilis* pCHlux101-P*iseA* and *B. subtilis* pCHlux101-P*iseA*∆*prkC* were grown to exponential phase and added to a 96-well plate with different x-fold MIC concentrations of the antibiotics. The assay was measured in TECAN plate reader for 3 h, measuring OD_600_ and luminescence (RLU) every 10 min. Mean values and their standard deviation (SD) obtained after 30 and 60 min are shown. The experimental cut-off is 200%. The higher values are presented in log_10_. Welch´s t-test was performed to compare the data sets with the respective parent strains with *prkC*. P < 0.001; ***P < 0.01; ** P < 0.05; *

The serine/threonine kinase PrkC was deleted in the P*iseA* and P*liaI* reporters in an attempt to optimise signal strength by eliminating the phosphorylation of WalR by PrkC. Whole genome sequencing confirmed the successful deletion and showed that both strains carried two additional point mutations, which, however, should not affect the promoters and regulons (Supplemental Table S6). A twofold increase in the P*iseA* signal was detected in the presence of vancomycin after 30 and 60 min, but not with the other tested control antibiotics (Fig. 5, Supplemental Figure S5). Conversely, P*liaI* expression (Fig. 5) decreased significantly in the absence of *prkC* 30 and 60 min after the addition of vancomycin and teicoplanin (Fig. 5). No significant differences in the expression of both *prkC* mutants compared to the wild type were observed following treatment with penicillin G, meropenem or fosfomycin after 30 min (Fig. 5); however, signal strength increased with the mutant strains with meropenem after 60 min and for penicillin and teicoplanin after 90 or 100 min, i.e. after entry into stationary phase (Supplemental Figure S6).

### Drug screening identifies possible inhibitors of cell wall biosynthesis

In order to evaluate the applicability of the complementary reporter system for compounds with unknown modes of action, natural products from myxobacterial and fungal sources were screened using both the P*liaI* and P*iseA* reporter strains. As LiaRS and WalRK represent different cell envelope stress response systems, variations in the activation patterns of the reporters may offer further insights into the cellular processes affected by these compounds.

**Fig. 6:**
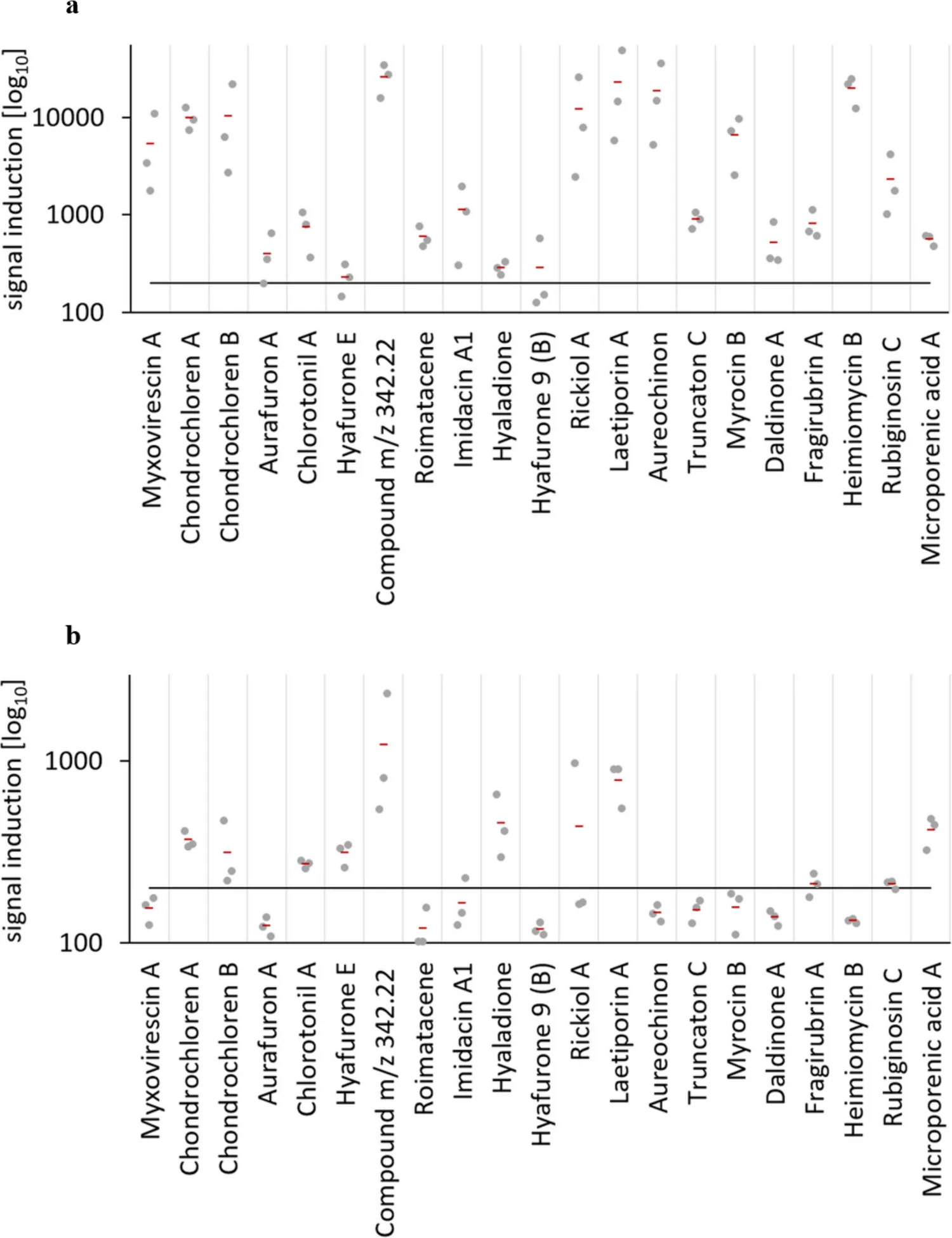
Maximal upregulation of P*liaI* and P*iseA* in presence of natural products of the HIPS. *B. subtilis* pCHlux101-P*liaI* (**a**), and *B. subtilis* pCHlux101-P*iseA* (**b**) were grown to exponential phase and added to a 96-well plate with different concentrations of the natural products. The assay was measured in TECAN plate reader for 3 h, measuring OD_600_ and luminescence (RLU) every 10 min. Data represent three biological replicates shown as grey dots, with the corresponding mean indicated by a red line

Figure 6 displays the natural compounds that showed an effect with the reporter strains *B. subtilis* pCHlux101-P*liaI* (**a**) and *B. subtilis* pCHlux101-P*iseA* (**b**) (Supplemental Table S7).

Several compounds, including chondrochloren A/B (Jansen et al. 2003; Weissman and Müller 2010), chlorotonil A (Bublitz et al. 2023; Deschner et al. 2025), compound *m/z* 342.22 and laetiporin A (Chepkirui et al. 2017) showed a vancomycin-like pattern (Fig. 3) with upregulation of P*liaI* and P*iseA*. In vitro testing with the purified WalK and WalR proteins showed that none of these substances directly interfered with kinase activity (Supplemental Figure S7, cropped version; uncropped gel image provided in a separate supplementary file), suggesting that these compounds affect cell wall homeostasis or target other cellular processes associated with cell wall biosynthesis. For compound *m/z* 342.22 and chlorotonil A, the P*iseA/prkC* deletion mutant also showed a higher sensitivity (Supplemental Figure S8).

In contrast, hyafurone E (Okanya et al. 2014) exhibited a distinct reporter profile, inducing P*iseA* expression while only weakly affecting P*liaI* expression (see Fig. 6). Hyafurone compounds have previously been reported to exhibit only weak antibacterial activity, with MIC values above 67 µg/mL against tested bacterial strains (Okanya et al. 2014). Since antibacterial activity against *B. subtilis* could not be detected at the highest available concentration (25.24 µg/mL), the observed P*iseA* induction cannot directly be correlated with growth inhibition in this organism. However, this differential response underscores the value of the WalRK-dependent reporter system as a complementary tool, as it enables the identification of compounds with limited activation of the LiaRS response based on their effect on WalRK-regulated promoters.

Although hyafurone E activated the P*iseA* reporter, subsequent in vitro analysis using purified WalK and WalR proteins did not show a pronounced inhibition of WalRK activity (Supplemental Fig. 7). Therefore, the observed reporter response is probably due to an indirect effect on cell envelope homeostasis rather than direct inhibition of the WalRK system. The cytotoxic cyclohexadiene-dione hyaladione (Okanya et al. 2012) also induced P*iseA* expression above the defined threshold, but was not analysed further in subsequent assays.

## Discussion

### Validation of the WalRK-dependent reporter strains

Due to its essential role in maintaining bacterial survival, cell wall biosynthesis represents a promising target for antibiotics (Wolf and Mascher 2016). However, increasing antimicrobial resistance, including resistance against established cell wall-targeting antibiotics such as glycopeptides and β-lactams, highlights the urgent need for novel antibacterial compounds and targets. Screening methods that identify the modes of action of new, potential antibiotics, such as cell wall biosynthesis inhibitors, are widely used and essential. Various reporter systems have been published to assess the initial mode of action of a compound (Sun et al. 2002; Fischer et al. 2004; Urban et al. 2007; Wex et al. 2021). This study established and validated a set of *B. subtilis* reporter strains to monitor the activity of the essential WalRK system in response to known antibiotics and natural products. WalRK-dependent promoters, including those of the *iseA* and *ssaA* genes, were fused to *luxCDABE* based on the plasmid of the P*liaI* reporter system of Kobras et al. (2017).

The reporter strains with the promoters of *iseA* and *ssaA* successfully demonstrated a WalRK growth phase dependent expression pattern (Fig. 2), The P*iseA* reporter with low activity in the exponential phase and high activity in the stationary phase exhibited the reverse profile of the P*ssaA* reporter (Fabret and Hoch 1998). As expected, the expression of P*iseA* changed in the presence of cell wall biosynthesis inhibitors including glycopeptides, β-lactam antibiotics, daptomycin, tunicamycin and fosfomycin. This is essential because the continued activity of autolysins would be lethal during the inhibition of cell wall biosynthesis. While additional regulatory influences on promoter activity cannot be completely excluded, the selective induction of P*iseA* by cell wall biosynthesis inhibitors supports its suitability as a reporter for WalRK-dependent responses. No significant reporter responses were observed with antibiotics targeting protein, RNA, or DNA biosynthesis nor with colistin targeting membrane integrity (Fig. 4). This confirms that the WalRK system perceives imbalanced and disrupted peptidoglycan synthesis and adapts autolysin transcription (Dobihal et al. 2019). As mentioned above, the DNA binding motifs between *B. subtilis* and *S. aureus* show a high degree of identity (Dubrac and Msadek 2004). These observations were supported by the recognition of the *S. aureus ssaA* promoter by WalR in *B. subtilis*, and represent the strong conservation of WalR DNA-binding motifs in Gram-positive bacteria with low G + C content (Howell et al. 2003; Dubrac and Msadek 2004). We originally had included the promoter sequence of *ssaA* in order to investigate whether it is possible to use a promoter sequence that is probably not recognized by and independent of other *B. subtilis* regulatory proteins or sigma factors. In addition, P*ssaA* represents a promoter that is only activated by WalR, since the knockout of WalR in *S. aureus* is lethal, unless *ssaA* (or *lytM*) is put under control of another promoter which shows, that transcription of these genes—at least in *S. aureus*—is only activated by WalR (Dubrac and Msadek 2004; Delaune et al. 2011). In contrast, the essential autolysin genes of *B. subtilis*, *cwlO* and *lytE* (Bisicchia et al. 2007) are also under control of SigI (Tseng et al. 2011; Salzberg et al. 2013).

However, since a low signal strength of the P*ssaA* reporter was observed with all the tested antibiotics, this decrease cannot be considered specific for impaired WalRK signalling and is more likely to reflect general physiological effects such as growth inhibition, reduced metabolic activity, toxic effects or inhibition of translation or transcription. A limitation of this interpretation is that growth inhibition was inferred from OD_600_ measurements, which reflect changes in cell density but do not directly assess viability. Therefore, reduced reporter signals may be attributable to bacteriostatic effects or cell death, depending on the mode of action of the tested compound. Consequently, the P*ssaA* reporter is primarily suitable for detecting activators of WalK activity (Cheng et al. 2024).

### The WalRK reporters complement the P*liaI* reporter system

Comparison of the WalRK-dependent reporters with the P*liaI* reporter revealed notable differences in the expression levels of the reporters in presence of cell wall biosynthesis inhibitors (Fig. 3). As previously published, the *liaI* promoter was strongly induced by antibiotics that interfere with the lipid II cycle, such as vancomycin, mersacidin, nisin, and tunicamycin (Mascher et al. 2004; Jordan et al. 2006). Although the *liaI* promoter showed higher maximal fold induction than the *iseA* promoter, the P*iseA* signal was reproducible and detectable across glycopeptide treatment (Cao et al. 2002) representing a decreased WalRK activity. While the P*liaI* reporter provided a highly sensitive readout for general cell envelope stress and especially the lipid II cycle, the P*liaI* reporter did not respond to β-lactam antibiotics such as penicillin G and meropenem or fosfomycin, which, however, were detected with P*iseA* (Fig. 3; Mascher et al. 2004). This reflects the distinct physiological functions of the WalRK and LiaRS systems. WalRK may sense a broader spectrum of antibiotics that confer inhibition of peptidoglycan synthesis (Dobihal et al. 2019), whereas LiaRS primarily detects accumulation of lipid II and membrane stress (Jordan et al. 2006; Suntharalingam et al. 2009). These observations are consistent with previous studies that suggest that cell wall-targeting antibiotics, such as glycopeptides and β-lactams, may indirectly modulate WalRK activity via specific cell wall cleavage products (Cao et al. 2002; Dhiman et al. 2015; Dobihal et al. 2019). This process might be analogous to the signal pathway that leads to induction of AmpC in *Enterobacter* and *Citrobacter* (Jacobs et al. 1994). Other publications hypothesised that the WalKHI complex senses the functioning of the cell wall biosynthetic complex and cell division (Fukushima et al. 2011).

Taken together, these results demonstrate that the P*iseA* reporter strain complements the P*liaI* reporter. Combining these reporter strains enables the detection of a wide range of antibiotics that attack the cell envelope. In addition to β-lactams, the range of the *iseA* reporter also includes fosfomycin, an antibiotic that targets the early steps of peptidoglycan biosynthesis, and is broader than that of a former biosensor developed by Lautenschläger et al. (2020) which is limited to β-lactams only. This combination enhances the identification of cell wall inhibitors in screening assays for novel antibiotics and enables the simultaneous detection of lipid II-associated stress and peptidoglycan synthesis inhibition.

### The signal strength of the P*iseA* reporter strain increases in response to vancomycin and in stationary phase in *prkC* knockout strains

The eukaryotic-like Ser/Thr kinase (eSTK) PrkC in *B. subtilis* (called PknB in *S. aureus*) and its cognate eukaryotic-like Ser/Thr phosphatase (eSTP) PrpC (called Stp in *S. aureus*) also regulate the expression of WalRK-dependent genes, especially in the stationary phase, when WalK activity decreases (Libby et al. 2015; Hardt et al. 2017). In both organisms, PrkC/PknB phosphorylates WalR at residue Thr101, which increases the activity of WalR in *B. subtilis* and leads to the inhibition of *iseA* transcription primarily in the stationary phase (Libby et al. 2015). In *S. aureus*, the kinase senses the availability of lipid II by binding to the stem peptide of lipid II (Hardt et al. 2017). To optimise the reporter strains and eliminate the activating influence of PrkC on WalR activity in the reporter strains, *prkC* deletions were created within the P*iseA* and the P*liaI* reporters. In the knockout strains (Fig. 5), the expression of the negatively regulated promoter of *iseA* was increased in presence of vancomycin and some natural products, such as compound *m/z* 342.22 and chlorotonil A (Bublitz et al. 2023; Deschner et al. 2025). No data are currently available on the targets of compound *m/z* 342.22; however, Deschner et al. (2025) focus on the chlorotonil derivative dehalogenil and show that, in addition to its effect on the membrane, it is reported to target YbjG, an undecaprenyl pyrophosphate phosphatase involved in regeneration of the lipid carrier required for lipid II biosynthesis. In contrast, an enhancing effect of the deletion of *prkC* with β-lactam antibiotics such as penicillin G and meropenem in the exponential phase after 30 and 60 min was only observed for meropenem after 60 min (Fig. 5). The relatively moderate effect of the *prkC* deletion on P*iseA* expression after 30–60 min of incubation is consistent with PrkC functioning as an additional regulatory input into the WalRK system especially in the stationary phase, rather than as the primary mechanism controlling WalR activity (Libby et al. 2015). In accordance, the activity of P*iseA* in the presence of cell wall biosynthesis inhibitors increased upon entry into the stationary phase (90 or 100 min, Supplemental Figure S6), and therefore, the sensitivity of this reporter strain might be enhanced by longer incubation times. At first glance, a regulation that enhances WalR activity and autolysis in the stationary phase or presence of antibiotics seems irrational. However, an *S. aureus* strain with T101M mutation in WalR, that blocks phosphorylation, demonstrated that PrkC-mediated WalR phosphorylation is necessary for a peptidoglycan recycling pathway in the stationary phase (Tan et al. 2022).

Conversely, deleting *prkC* reduced the luminescence of the P*liaI* reporter in the presence of glycopeptides (Fig. 5). LiaS responds to lipid II-mediated stress (Mascher et al. 2004; Jordan et al. 2006). In addition, LiaS reacts to general membrane perturbations, such as detergents, ethanol, alkaline shock, and secretion stress. This reflects a phage shock protein (PSP)-like envelope stress response (Ravi et al. 2024). The mild phenotypes of *liaIH* mutants against antibiotics acting on cytoplasmic (e.g., fosfomycin) or extracellular (e.g., β-lactams) steps of cell wall biosynthesis demonstrate that stresses have to target the cytoplasmic membrane to trigger the LiaRS response (Domínguez-Escobar et al. 2014). The downregulation of P*liaI* in the *prkC* mutant reporter was unexpected and may reflect differences in the integration of P*liaI* and P*iseA* into the regulatory networks of *B. subtilis*. While P*iseA* represents a transcriptional output of WalRK activity – and in times of phosphate stress, also of the kinase PhoR, which is also able to phosphorylate WalR (Howell et al. 2006) -, P*liaI* reflects activation of the LiaRS envelope stress response, which integrates signals associated with membrane and lipid II stress. In addition, P*liaI* is repressed by the master regulator AbrB during the exponential phase and the AbrB binding motif is also present in the reporter strain (Jordan et al. 2007). Phosphorylated Spo0A will relieve the repression of P*liaI* at the transition into the stationary phase (Jordan et al. 2007). PrkC is also a master regulator with a phosphoproteome of more than 100 proteins, which, for example, includes the kinase KinC that is part of the phosphorelay activating Spo0A (Phillips and Strauch 2002; Ravikumar et al. 2014). Therefore, the downregulation of the P*liaI* reporter in the *prkC* mutant does not necessarily indicate a direct activation of P*liaI* by PrkC and to our knowledge, it has not been yet reported that LiaR or LiaS are substrates of PrkC in *B. subtilis* (Ravikumar et al. 2014; Zhang et al. 2018). Therefore, it remains to be investigated in further work whether PrkC influences LiaRS activity directly or indirectly.

### The combined use of the P*iseA*/P*ssaA* and P*liaI* reporter strains in natural product screening identifies antibiotics that affect the cell envelope

The WalRK-dependent reporters were combined with *liaI* to screen 66 myxobacterial and fungal natural products, to identify potential inhibitors of cell wall biosynthesis or compounds that target the kinase WalK directly. Of these, 21 compounds altered the expression of the reporters P*liaI* and P*iseA*. Several compounds displayed vancomycin-like patterns (Fig. 3), characterised by upregulation of P*liaI* and P*iseA*. Notably, chondrochloren A and B, chlorotonil A, compound *m/z* 342.22, and laetiporin A exhibited this pattern, suggesting that they interfere with cell wall integrity, although the precise mode of action of most compounds remains uncharacterised.

Some of the compounds have already been described as having antibacterial activity. For example, chondrochlorens A and B inhibit the growth of *B. subtilis*, though no direct interaction with the cell wall has been reported (Jansen et al. 2003; Weissman and Müller 2010). In contrast, chlorotonil A not only targets the membrane by binding lipids and causing depolarisation, but also inhibits regeneration of the undecaprenyl-pyrophosphate phosphatase involved in regeneration of the lipid carrier required for lipid II biosynthesis (Bublitz et al. 2023; Deschner et al. 2025).

In theory, upregulation of P*iseA* could also indicate a direct inhibition of WalK activity by the antibiotics. However, in vitro testing for direct effects of the compounds on the in vitro activity of purified WalK did not reveal significant changes in WalK activity (Supplemental Figure S7), indicating that the observed reporter responses primarily reflect cellular perception of cell wall biosynthesis inhibition. Therefore, investigating the P*iseA* reporter using published WalRK inhibitors is an interesting approach and will be in the focus of further studies. This approach would determine if the reporters can detect direct inhibition of WalRK, which would enhance their utility in future antibiotic screens and discovery efforts.

In conclusion, these results demonstrate that WalRK-dependent reporters successfully complement P*liaI*, capturing a broader spectrum of cell wall biosynthesis inhibition. While they do not provide evidence of a direct interaction between the antibiotic and WalK, they allow for the rapid identification of candidates for further mechanistic studies. Notably, the reporter patterns that were observed during the validation of known antibiotics were also observed with natural products. This confirms that these reporters are sensitive indicators of cell wall-related stress and valuable tools for screening novel compounds.

## Supplementary Information

Below is the link to the electronic supplementary material.ESM 1(PDF 1.48 MB)ESM 2(PDF 105 KB)

## Data Availability

The sequencing datasets generated during this study have been deposited in the NCBI repository under BioProject PRJNA1440371 (BioSample IDs SAMN56611315, SAMN56611316). All other data supporting the findings of this study are included within the main manuscript and its supplementary information files.
