# Supplementary material for: Construction of a novel reporter strain indicating inhibition of cell wall biosynthesis
Source: Appl Microbiol Biotechnol. 2026 Aug 28;110:260. doi: 10.1007/s00253-026-14013-7 (PMC13525142; doi:10.1007/s00253-026-14013-7)
Supplement: Supplementary file 1 [file 253_2026_14013_MOESM1_ESM.pdf]

Construction of a novel reporter strain indicating inhibition of cell wall biosynthesis – Supplemental

Applied Microbiology and Biotechnology

Maike Laura Karcher[1*], Annette Siskowski[1], Chantal Müller[1], Laura Engel[1], Vincent Franz[1], Susanne H. Kirsch[3,4], Birthe Sandargo[4,5], Tanja Schneider[2], Gabriele Bierbaum[1]

1) University Hospital Bonn, Bonn, Germany, Institute of Medical Microbiology, Immunology and Parasitology

2) Institute for Pharmaceutical Microbiology, University Hospital Bonn, University of Bonn, 53115 Bonn, Germany

3) Helmholtz Centre for Infection Research (HZI), Helmholtz Institute for Pharmaceutical Research Saarland (HIPS), 66123 Saarbrücken, Germany

4) German Centre for Infection Research (DZIF), Partner Site Hannover-Braunschweig, 38124 Braunschweig, Germany.

5) Helmholtz Centre for Infection Research (HZI), 38124 Braunschweig, Germany.

*Corresponding author

Maike Laura Karcher (s74mkarc@uni-bonn.de); ORCID: 0009-0000-9804-8173

Institute of Medical Microbiology, Immunology and Parasitology,

University Hospital Bonn

Venusberg-Campus 1

53127 Bonn

Supplementary Data

Figures

Figure S1: Luminescence signal (RLU) over time and associated growth curves of WalRK-dependent reporter strains: P*pdaC* (a) and P*ykvT* (b) in *B. subtilis* 168, representing WalRK activity. *B. subtilis* pCHlux101-P*pdaC*, *B. subtilis* pCHlux101-P*ykvT* were grown to exponential phase and added to a 96-well plate. The assay was measured in a TECAN plate reader for 3 hours, measuring OD_{600} (blue) and luminescence (RLU) every 10 minutes (red).

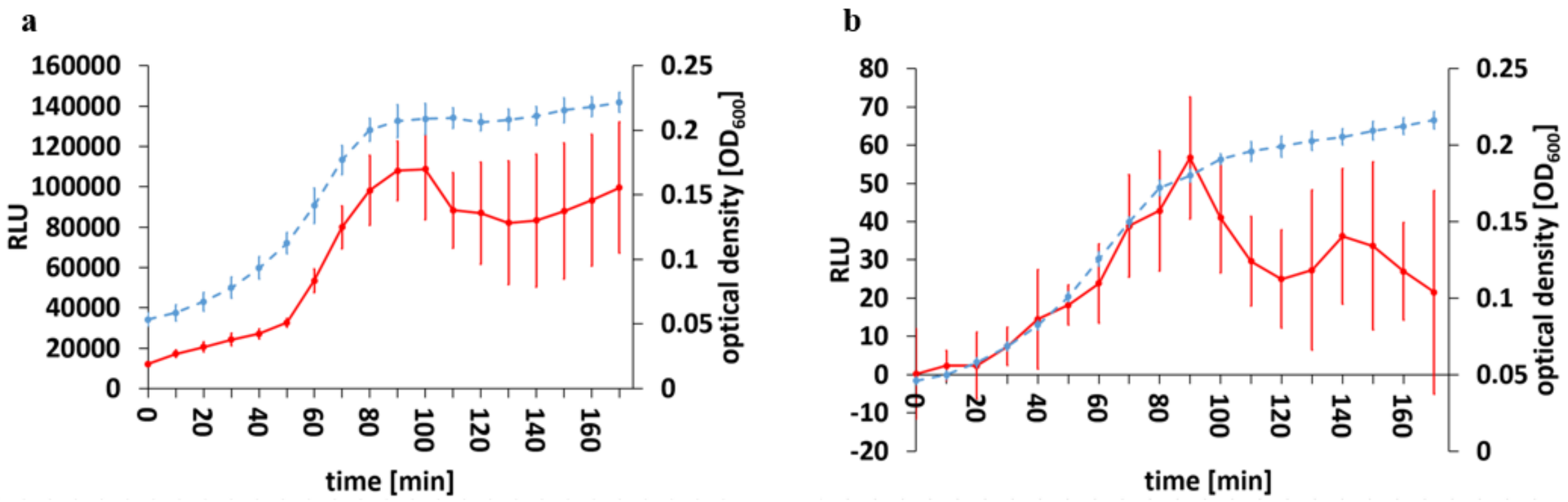

Figure S2: Expression levels represented as signal induction in percent [%] over time (left) and associated growth curves (right) of *liaI* (a) and the WalRK-dependent genes *iseA* (b) and *ssaA* (c) in *B. subtilis* 168 in presence of vancomycin. *B. subtilis* pCHlux101-P*liaI*, *B. subtilis* pCHlux101-P*iseA*, *B. subtilis* pCHlux101-P*ssaA* were grown to exponential phase and added to a 96-well plate with different x-fold MIC concentrations of vancomycin. The assay was measured in TECAN plate reader for 3 hours, measuring OD_{600} and luminescence (RLU) every 10 minutes. Welch´s t-test was performed to compare the data sets to the growth control. P<0.001; ***, P<0.01; **, P<0.05; *.

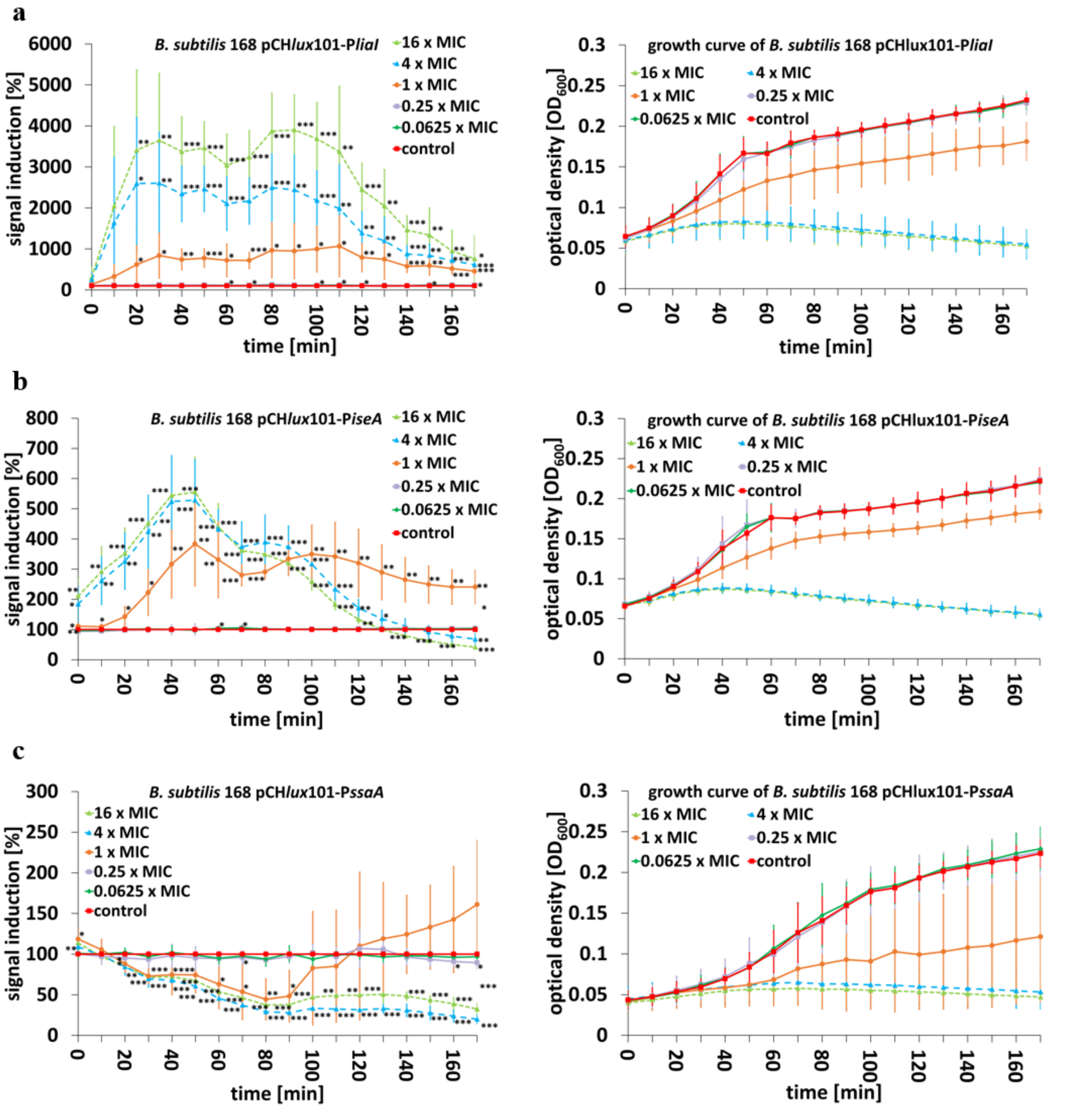

Figure S3: Expression levels of P*pdaC* (a) and P*ykvt* (b) represented as signal induction in percent [%] applied against MIC-fold of vancomycin normalized to growth control (red). *B. subtilis* pCHlux101-P*pdaC*, *B. subtilis* pCHlux101-P*ykvT* were grown to exponential phase and added to a 96-well plate with different x-fold MIC concentrations of vancomycin. The assay was measured in TECAN plate reader for 3 hours, measuring OD_{600} and luminescence (RLU) every 10 minutes. Welch´s t-test was performed to compare the data sets to the growth control. P<0.001; ***, P<0.01; **, P<0.05; *.

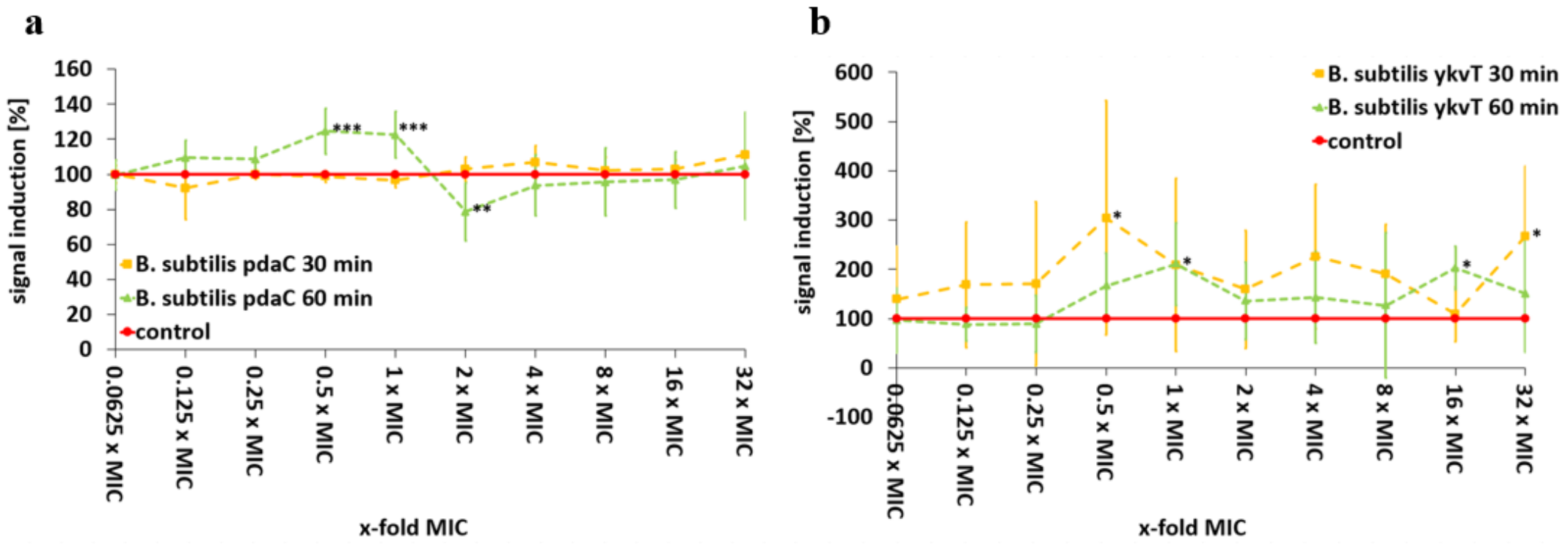

Figure S4: Expression levels of *liaI* (a), *iseA* (b) and *ssaA* (c) represented as signal induction in percent [%] applied against MIC-fold of kanamycin normalized to growth control (red). *B. subtilis* pCHlux101-P*liaI*, *B. subtilis* pCHlux101-P*iseA*, *B. subtilis* pCHlux101-P*ssaA* were grown to exponential phase and added to a 96-well plate with different x-fold MIC concentrations of vancomycin. The assay was measured in TECAN plate reader for 3 hours, measuring OD_{600} and luminescence (RLU) every 10 minutes. Welch´s t-test was performed to compare the data sets to the growth control. P<0.001; ***, P<0.01; **, P<0.05; *.

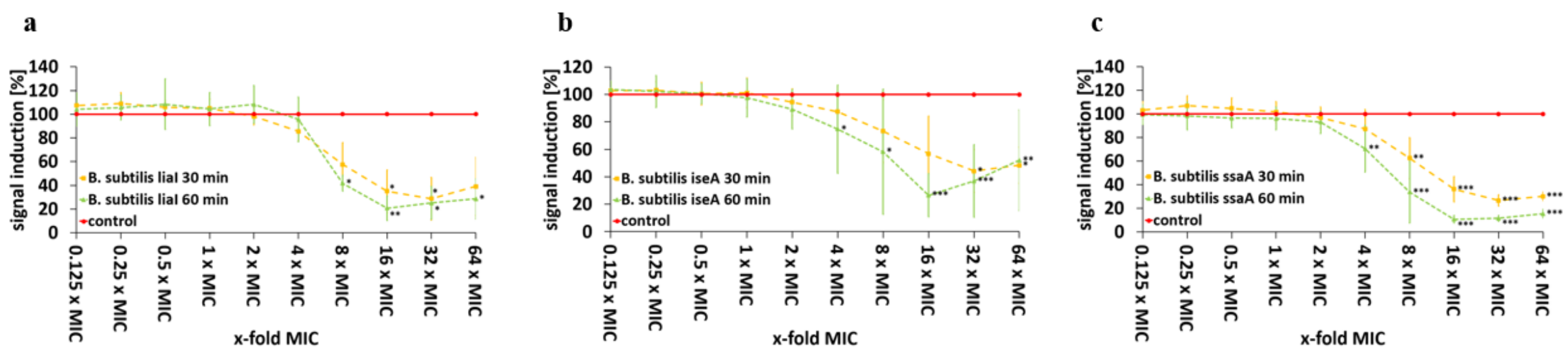

Figure S5: Expression levels of *liaI* (a) and *iseA* (b) in presence of vancomycin in the different variants of the reporter strains *B. subtilis* pCHlux101-P*liaI*, *B. subtilis* pCHlux101-P*liaI*Δ*prkC*, *B. subtilis* pCHlux101-P*iseA* and *B. subtilis* pCHlux101-P*iseA*Δ*prkC*. The gene expression was represented as signal induction in percent [%] applied against MIC-fold of vancomycin. The cells were grown to exponential phase and added to a 96-well plate with different x-fold MIC concentrations of vancomycin. The assay was measured in TECAN plate reader for 3 hours, measuring OD_{600} and luminescence (RLU) every 10 minutes. Welch´s t-test was performed to compare the data sets of the mutant to the wild type reporter strains. P<0.001; ***, P<0.01; **, P<0.05; *.

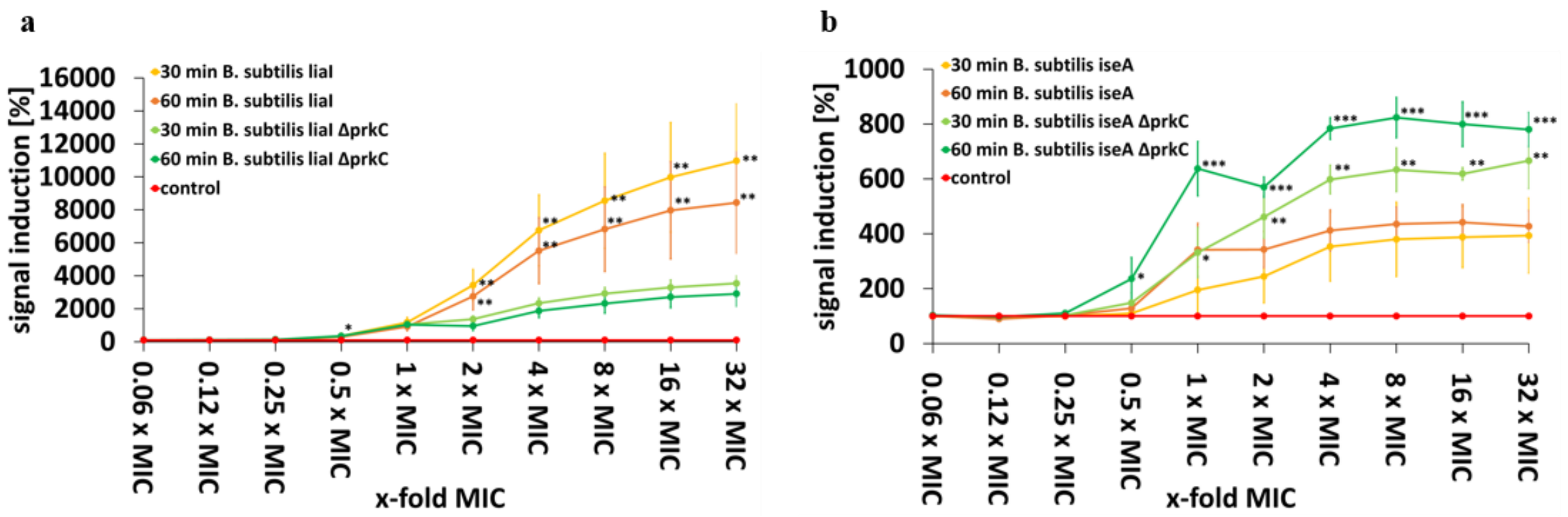

Figure S6: Maximal upregulation of P*liaI* and P*iseA* compared to *prkC* mutants in presence of various antibiotics, after 90 min and 100 min. *B. subtilis* pCHlux101-P*liaI*, *B. subtilis* pCHlux101-P*liaI*Δ*prkC*, *B. subtilis* pCHlux101-P*iseA* and *B. subtilis* pCHlux101-P*iseA*Δ*prkC* were grown to exponential phase and added to a 96-well plate with different x-fold MIC concentrations of the antibiotics. The assay was measured in TECAN plate reader for 3 hours, measuring OD_{600} and luminescence (RLU) every 10 minutes. Mean values and their standard deviation (SD) obtained after 90 and 100 minutes are shown. The experimental cut-off is 200% (red line). The higher values are presented in log_{10}.

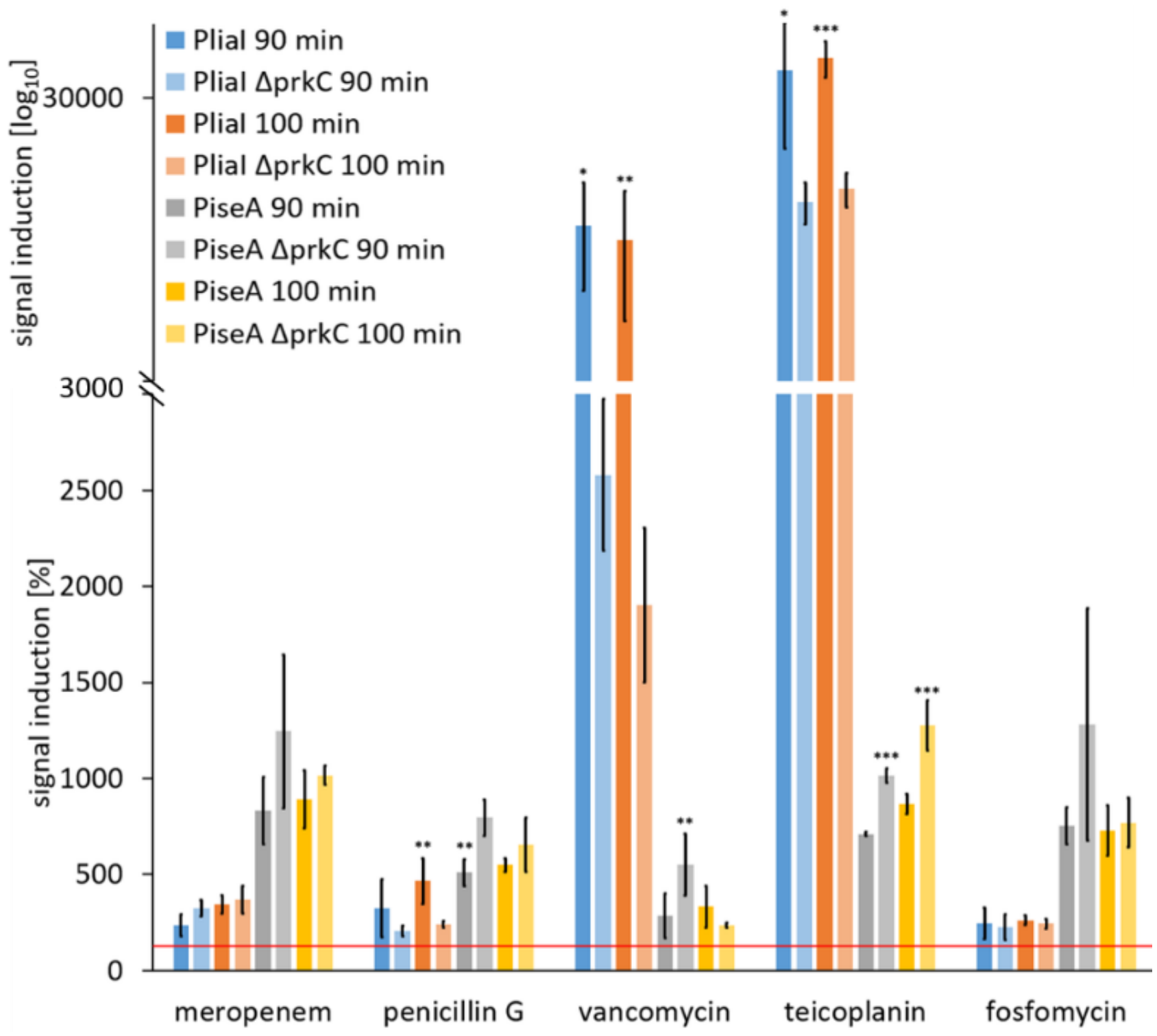

Figure S7: Transphosphorylation of WalRK in presence of a few selected natural products that looked interesting *in vivo*. The compounds were used in highest possible concentration, a negative control (WalRK without ATP), a positive control (WalRK with ATP) and a DMSO (WalRK with DMSO) control were performed. The identities of the natural products corresponding to the individual lanes are provided in Supplemental Table S7. The gel showed dephosphorylated WalK (highest band), phosphorylated WalR (middle band) and dephosphorylated WalR (lowest band).

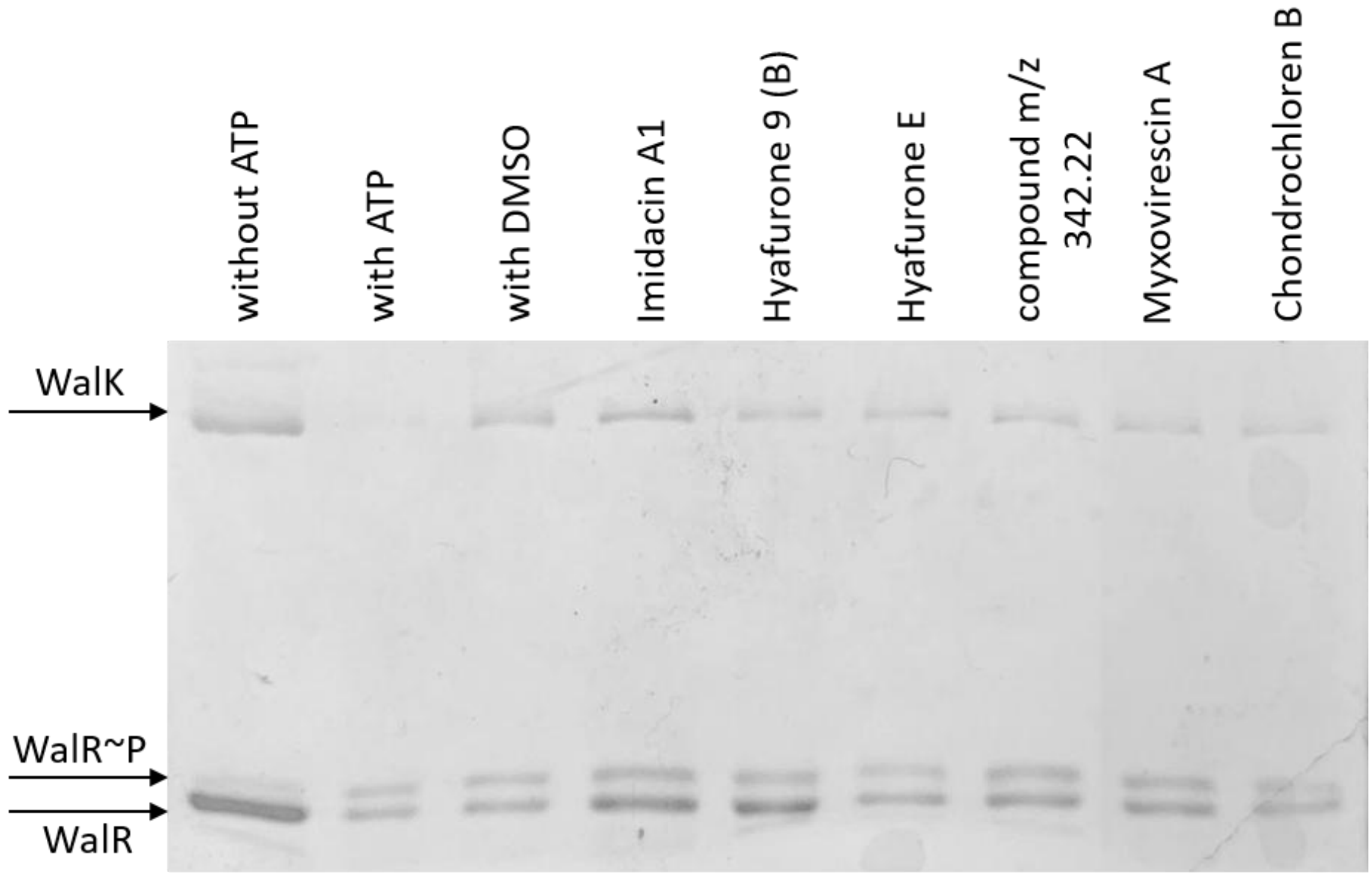

Figure S8: Maximal induction of P*iseA* in presence of chlorotonil A (a) and compound *m/z* 342.22 (b) in the different variants of the reporter strains *B. subtilis* pCHlux101-P*iseA* and *B. subtilis* pCHlux101-P*iseA*Δ*prkC*. The values of P*iseA* and P*iseA* Δ*prkC* were normalized to the maximal induction of P*iseA* Δ*prkC* independent of time and concentration. The normalized expression was represented as signal induction in percent [%] applied against compound concentration. The cells were grown to exponential phase and added to a 96-well plate with different compound concentrations. The assay was measured in TECAN plate reader for 3 hours, measuring OD_{600} and luminescence (RLU) every 10 minutes.

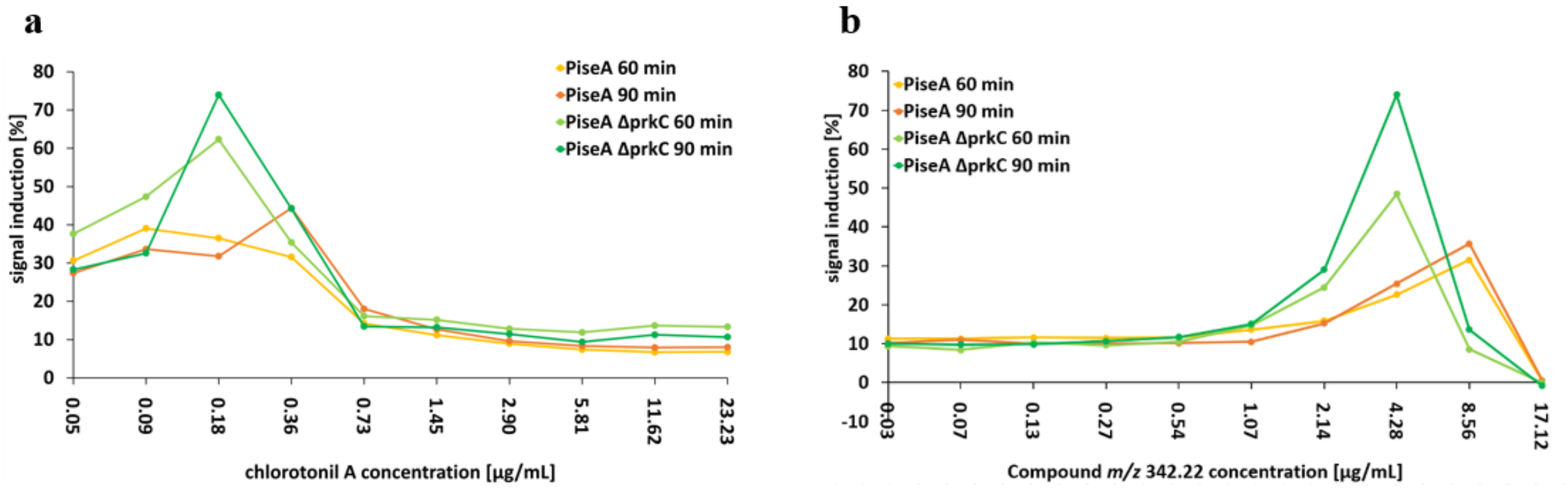

Tables

Table S1: Oligonucleotides used in this study.

Primer	Sequence 5'-3'and restriction sites	T_M [°C]	Reference
pBs3Clux-for	GAGCGTAGCGAAAAATCC	60.0	This work
pBs3Clux-rev	GAAATGATGCTCCAGTAACC	60.0	This work
iseA_for_NotI	AAAGCGGCCGCGTAACATGTTATTATTCATCACTGC	60.0	This work
iseA_rev_SalI	AAAGTCGACCTCTTTCTCTTATGTTACAAATGTTAC	60.0	This work
ykvT_for_NotI	AAAGCGGCCGCGTGAGAAGCCTTTTTATCATATATG	59.0	This work
ykvT_rev_SalI	AAAGTCGACCTTCATAACCTTAACAAGAGATCC	59.0	This work
pdaC_for_NotI	AAAGCGGCCGCCCATATTCCTGATGCTTAAGTTG	61.0	This work
pdaC_rev_SalI	AAAGTCGACGCCATTTCGATTTACTCTTCATC	61.0	This work
ssaA_for_NotI	AAAGCGGCCGCCTGATTAAAAGTCCTATATTATGCAC	60.0	This work
ssaA_rev_SalI	AAAGTCGACATATTTTCGTAATGTCCGTGTG	60.0	This work
left arm_fwd	AGACGCTGAACATCATCCGC	63	This work
left arm_rev with pre-sequence	aggtggcacttttcggggaaTGATCGAGGCTTGATGCGGA	64	This work
ermB with pre-seuence_fwd	tccgcatcaagcctcgatcaTTCCCCGAAAAGTGCCACCT	64	This work
ermB with pre-sequence_rev	gcacttgaagctcgtcccctCTCACTGCAGAGATCCCCGG	64	This work
right arm_fwd	ccggggatctctgcagtgagAGGGGACGAGCTTCAAGTGC	64	This work
right arm_rev	GCCGTGCTGAAAGAAGGCTG	64	This work

Table S2: Bacterial strains used in this study.

Strain	Relevant characteristic (s)	Reference (s) / source
B. subtilis 168 pCH*lux*101-P*liaI*	genomically integrated pCH*lux*101-P*liaI* plasmid in *sacA*, with the promoter of *liaI*	(Kobras et al. 2017)
B. subtilis 168 pCH*lux*101-P*iseA*	genomically integrated pCH*lux*101-P*iseA* plasmid in *sacA*, with the promoter of *iseA*	This work
B. subtilis 168 pCH*lux*101-P*ykvT*	genomically integrated pCH*lux*101-P*ykvT* plasmid in *sacA*, with the promoter of *ykvT*	This work
B. subtilis 168 pCH*lux*101-P*pdaC*	genomically integrated pCH*lux*101-P*pdaC* plasmid in *sacA*, with the promoter of *pdaC*	This work
B. subtilis 168 pCH*lux*101-P*ssaA*	genomically integrated pCH*lux*101-P*ssaA* plasmid in *sacA*, with the promoter of *ssaA*	This work
B. subtilis 168 pCH*lux*101-P*liaI*Δ*prkC*	genomically integrated pCH*lux*101-P*liaI* plasmid in *sacA*, with the promoter of *liaI*; knockout of *prkC*, integration of *ermB*	This work
B. subtilis 168 pCH*lux*101-P*iseA*Δ*prkC*	genomically integrated pCH*lux*101-P*iseA* plasmid in *sacA*, with the promoter of *iseA*; knockout of *prkC*, integration of *ermB*	This work
E. coli BL21(DE3)pREP4groESL(MT) pET22bΔ*pelB*-*vicR*	pET22bΔ*pelB* with *vicR* of *S. aureus* SA137/93A	(Türck and Bierbaum 2012)
E. coli C43(DE3)pREP4groESL(MT) pET22bΔ*pelB*-*vicK*	pET22bΔ*pelB* with *vicK* of *S. aureus* SA137/93A	(Türck and Bierbaum 2012)

Table S3: MICs measured in this study for the reporter strains *B. subtilis* pCHlux101-P*liaI*, *B. subtilis* pCHlux101-P*iseA*, *B. subtilis* pCHlux101-P*ssaA*. For antibiotics tested with Δ*prkC* reporter strains, MIC values were identical to those of the corresponding wild-type strains. Not detectable (n.d).

Antibiotic	*B. subtilis pCHlux101-PliaI*	*B. subtilis pCHlux101-PiseA*	*B. subtilis pCHlux101-PssaA*
Vancomycin	0.25 µg/mL	0.25 µg/mL	0.25 µg/mL
Meropenem	0.125 µg/mL	0.125 µg/mL	0.125 µg/mL
Penicillin G	0.063 µg/mL	0.063 µg/mL	0.031 µg/mL
Mersacidin	32 µg/mL	32 µg/mL	32 µg/mL
Tunicamycin	n.d	n.d	n.d
Nisin	2 µg/mL	2 µg/mL	4 µg/mL
Colistin	16 µg/mL	16 µg/mL	16 µg/mL
Fosfomycin	20 µg/mL	20 µg/mL	20 µg/mL
Kanamycin	0.125 µg/mL	0.125 µg/mL	0.125 µg/mL
Erythromycin	0.125 µg/mL	0.125 µg/mL	0.125 µg/mL
Rifampicin	0.007 µg/mL	0.007 µg/mL	0.007 µg/mL
Ciprofloxacin	0.5 µg/mL	1 µg/mL	0.5 µg/mL
Teicoplanin	0.031 µg/mL	0.031 µg/mL	-
Daptomycin	1 µg/mL	1 µg/mL	1 µg/mL

Table S4: Basal expression in RLU (relative light units) over time of the promoters *liaI*, *iseA* and *ssaA* in presence of 32 x MIC vancomycin and without treatment

Time [min]	P*liaI* growth control	P*iseA* growth control	P*ssaA* growth control	P*liaI* 32 x MIC	P*iseA* 32 x MIC	P*ssaA* 32 x MIC
0	159	3273	21336	272	7489	22774
10	201	5328	28303	2175	14268	25494
20	290	7012	34423	6359	21094	25549
30	429	7675	42995	9667	26029	26252
40	549	8670	46939	10653	27697	27432
50	572	9704	60653	10210	26496	27868
60	656	12111	86584	9196	23417	26684
70	521	12687	109993	7724	19674	24321
80	416	11752	134766	6278	16045	21135
90	355	10788	128595	5004	12738	17251
100	326	11388	98883	3850	10130	14125
110	327	13626	80706	2899	8068	11277
120	344	16317	71301	2238	6355	8955
130	350	18331	57723	1669	5073	7234
140	331	19991	52187	1283	3997	5908
150	318	21567	51004	947	3206	4785
160	316	23290	51214	665	2558	3855
170	334	25004	52964	459	2053	3154

Table S5: Maximal upregulation of *liaI*/*iseA* and downregulation of *ssaA*. The concentration that induced this up- or downregulation is indicated below.

		Induction of promoter					
antibiotic		P*liaI*		P*iseA*		P*ssaA*	
		30 min	60 min	30 min	60 min	30 min	60 min
vancomycin	Up/down regulation	4000%	3200%	450%	430%	68%	37%
	Concentration (x-fold MIC)	32	32	32	32	2	2
meropenem	Up/down regulation	144%	179%	459%	844%	66%	17%
	Concentration (x-fold MIC)	1	1	4	2	8	8
penicillin G	Up/down regulation	167%	179%	240%	404%	74%	29%
	Concentration (x-fold MIC)	254	254	127	127	32	2032
mersacidin	Up/down regulation	2791%	6516%	199%	201%	26%	22%
	Concentration (x-fold MIC)	4	0.25	4	0.125	16	16
tunicamycin	Up/down regulation	705%	1014%	170%	287%	81%	61%
	Concentration (µg/mL)	8 µg/mL	8 µg/mL	2 µg/mL	4 µg/mL	16 µg/mL	16 µg/mL
nisin	Up/down regulation	1592%	2871%	106%	111%	-3%	-17%
	Concentration (x-fold MIC)	1	1	0.5	0.5	2	2
colistin	Up/down regulation	109%	107%	102%	137%	39%	33%
	Concentration (x-fold MIC)	0.015	0.015	0.015	0.5	4	4
fosfomycin	Up/down regulation	118%	133%	173%	610%	92%	64%
	Concentration (x-fold MIC)	64	64	64	64	32	32
kanamycin	Up/down regulation	108%	108%	103%	103%	27%	11%
	Concentration (x-fold MIC)	0.25	0.5	0.25	0.125	32	16
erythromycin	Up/down regulation	111%	233%	118%	102%	24%	13%
	Concentration (x-fold MIC)	1	1	0.5	2	64	64
rifampicin	Up/down regulation	56%	31%	80%	83%	51%	23%
	Concentration (x-fold MIC)	8	8	1	1	571	571
ciprofloxacin	Up/down regulation	117%	141%	103%	124%	68%	49%

	Concentration (x-fold MIC)	0.062	0.062	0.5	0.5	8	8
teicoplanin	Up/down regulation<br>Concentration (x-fold MIC)	34440%<br>10	26846%<br>10	683%<br>80	840%<br>10	-	-
daptomycin	Up/down regulation<br>Concentration (x-fold MIC)	13619%<br>2	50689%<br>2	152%<br>8	243%<br>4	52%<br>16	43%<br>16

Table S6: Detected mutations in the Δ*prkC* mutants *liaI*/*iseA* compared to the wt reporter strains and the corresponding effects on the sequence

Strain	Position/gene	Reference Allele	Variant Allele	Variant Frequency	Sequence effect
B. subtilis pCH*lux*101-P*liaI* Δ*prkC*	171679/*rrnG*-16S rRNA	TA	T	83.64%	
	2142916/*yodQ* (putative deacylase)	CT	C	40.24 %	Frameshift, different amino acids, modifies protein
B. subtilis pCH*lux*101-*PiseA* Δ*prkC*	166704/rrnH-16S rRNA	C	A	100%	
	2504205/*ptb* (phosphate butyryltransferase)	G	A	100%	Codon CAA encoding glutamine to TAA encoding stop codon

Following page:

Table S7: 21 compounds that were analysed in a natural product screen from DZIF showed changes in *liaI* and *iseA* promoter expression with "+" representing an increased gene expression compared to the growth control and "-" showing a lower expression compared to the growth control and "=" indicates that no change in expression compared to the control was recorded. Additionally, *in vitro* and published results are presented.

Natural products	Expression of *liaI*		Expression of *iseA*		*in vitro* results	Effect according to literature
	30 min	60 min	30 min	60 min		
Myxovirescin A	+	+	=	=	no effect	cell wall biosynthesis inhibitor; targets type II signal peptidase LspA (Trowitzsch et al. 1982; Weissman and Müller 2010; Xiao et al. 2012)
Chondrochloren A	+	+	+	+	no effect	antibacterial effect against *M. luteus*, *B. subtilis*, *S. aureus* (Jansen et al. 2003; Weissman and Müller 2010)
Chondrochloren B	+	+	-	+	no effect	antibacterial effect against *M. luteus*, *B. subtilis*, *S. aureus* (Jansen et al. 2003; Weissman and Müller 2010)
Aurafuron A	=	+	-	-	no effect	weakly active against Gram-positive bacteria (Weissman and Müller 2010)
Chlorotonil A	=	+	=	+	no effect	targets membrane by binding to lipids; depolarization of the membrane (Bublitz et al. 2023; Deschner et al. 2025)
Hymeron/Hyafurone E	-	-	=	+	no effect	weak antibacterial activity against various bacterial strains (Okanya et al. 2014)
Compound *m/z* 342.22	+	+	=	+	no effect	-
Roimatacen	+	+	-	=	no effect	antibacterial effect against Gram-negative bacteria (Zander et al. 2011)
Imidacin A1	+	+	=	=	no effect	(Kostka et al. 2024)
Hyaladione	-	-	=	+	no effect	antibacterial effect against *S. aureus*; cytotoxic (Okanya et al. 2012)
Hyafurone B	=	+	-	-	no effect	weak antibacterial activity against various bacterial strains (Okanya et al. 2014)
Rickiol A	+	+	=	=	no effect	antibacterial effect against *S. aureus* (Surup et al. 2018)
Laetiporin A	+	+	+	+	no effect	cytotoxic effect; antimicrobial activity unknown (Chepkirui et al. 2017)
Aureoquinone	=	+	-	-	no effect	antibacterial effect against *B. subtilis* (Berg et al. 2000)
Truncatone C	+	+	=	=	no effect	- (Sudarman et al. 2016)
Myrocin B	=	=	-	-	no effect	antibacterial effect against *B. subtilis* (Hsu et al. 1988; Lehr et al. 2006)
Daldinone A	+	+	=	=	no effect	- (Quang et al. 2002)
Fragirubrin A	+	+	=	=	inhibition	slight inhibitory effect against *S. aureus* biofilm (Becker et al. 2021b; Becker et al. 2021a)
Heimiomycin B	+	+	-	-	inhibition	antibacterial effect against *B. subtilis* and *S. aureus* (Cheng et al. 2020; Pfütze et al. 2023)
Rubiginosin C	+	+	=	=	inhibition	- (Zeng et al. 2023)
Microporenic acid	=	=	=	=	no effect	Moderate activity against *M. luteus*; dose-dependent inhibition of biofilm formation by *S. aureus*; weak cytotoxicity activity; fungistatic growth stop (Chepkirui et al. 2018)

References

Becker K, Kuhnert E, Cox RJ, Surup F (2021a) Azaphilone Pigments from Hypoxylon rubiginosum and H. texense : Absolute Configuration, Bioactivity, and Biosynthesis. Eur J Org Chem 2021:5094–5103. https://doi.org/10.1002/ejoc.202001661

Becker K, Pfütze S, Kuhnert E, Cox RJ, Stadler M, Surup F (2021b) Hybridorubrins A-D: Azaphilone Heterodimers from Stromata of Hypoxylon fragiforme and Insights into the Biosynthetic Machinery for Azaphilone Diversification. Chemistry 27:1438–1450. https://doi.org/10.1002/chem.202003215

Berg A, Görls H, Dörfelt H, Walther G, Schlegel B, Gräfe U (2000) Aureoquinone, a new protease inhibitor from Aureobasidium sp. J Antibiot (Tokyo) 53:1293–1295. https://doi.org/10.7164/antibiotics.53.1293

Bublitz A, Brauer M, Wagner S, Hofer W, Müsken M, Deschner F, Lesker TR, Neumann-Schaal M, Paul L-S, Nübel U, Bartel J, Kany AM, Zühlke D, Bernecker S, Jansen R, Sievers S, Riedel K, Herrmann J, Müller R, Fuchs TM, Strowig T (2023) The natural product chlorotonil A preserves colonization resistance and prevents relapsing *Clostridioides difficile* infection. Cell Host Microbe 31:734-750.e8. https://doi.org/10.1016/j.chom.2023.04.003

Cheng T, Chepkirui C, Decock C, Matasyoh JC, Stadler M (2020) Heimiomycins A-C and Calamenens from the African Basidiomycete Heimiomyces sp. Journal of Natural Products 83:2501–2507. https://doi.org/10.1021/acs.jnatprod.0c00486

Chepkirui C, Matasyoh JC, Decock C, Stadler M (2017) Two cytotoxic triterpenes from cultures of a Kenyan Laetiporus sp. (Basidiomycota). Phytochemistry Letters 20:106–110. https://doi.org/10.1016/j.phytol.2017.04.009

Chepkirui C, Yuyama KT, Wanga LA, Decock C, Matasyoh JC, Abraham W-R, Stadler M (2018) Microporenic Acids A–G, Biofilm Inhibitors, and Antimicrobial Agents from the Basidiomycete Microporus Species. Journal of Natural Products 81:778–784. https://doi.org/10.1021/acs.jnatprod.7b00764

Deschner F, Mostert D, Daniel J-M, Voltz A, Schneider DC, Khangholi N, Bartel J, Pessanha de Carvalho L, Brauer M, Gorelik TE, Kleeberg C, Risch T, Haeckl FJ, Herraiz Baní-tez L, Andreas

A, Kany AM, Jézéquel G, Hofer W, Müsken M, Held J, Bischoff M, Seemann R, Brötz-Oesterhelt H, Schneider T, Sieber S, Müller R, Herrmann J (2025) Natural products chlorotonils exert a complex antibacterial mechanism and address multiple targets. Cell Chemical Biology 32:586-602.e15. https://doi.org/10.1016/j.chembiol.2025.03.005

Hsu Y-H, Nakagawa M, Hirota A, Shima S, Nakayama M (1988) Structure of Myrocin B, a New Diterpene Antibiotic Produced by Myrothecium verrucaria. Agricultural and Biological Chemistry 52:1305–1307. https://doi.org/10.1080/00021369.1988.10868805

Jansen R, Kunze B, Reichenbach H, Höfle G (2003) Chondrochloren A and B, New β-Amino Styrenes from *Chondromyces crocatus* (Myxobacteria). Eur J Org Chem 2003:2684–2689. https://doi.org/10.1002/ejoc.200200699

Kobras CM, Mascher T, Gebhard S (2017) Application of a *Bacillus subtilis* Whole-Cell Biosensor (PliaI-lux) for the Identification of Cell Wall Active Antibacterial Compounds. Methods Mol Biol 1520:121–131. https://doi.org/10.1007/978-1-4939-6634-9_7

Kostka M, Krug D, Herrmann J, Dickschat JS, Meyer J, Müller R, Schulz S (2024) Identification by Synthesis: Imidacins, Urocanate-Derived Alkaloids from the Myxobacterium *Stigmatella aurantiaca*. Organic Letters 26:6359–6363. https://doi.org/10.1021/acs.orglett.4c02036

Lehr N-A, Meffert A, Antelo L, Sterner O, Anke H, Weber RWS (2006) Antiamoebins, myrocin B and the basis of antifungal antibiosis in the coprophilous fungus Stilbella erythrocephala (syn. S. fimetaria). FEMS Microbiol Ecol 55:105–112. https://doi.org/10.1111/j.1574-6941.2005.00007.x

Okanya PW, Mohr KI, Gerth K, Steinmetz H, Huch V, Jansen R, Müller R (2012) Hyaladione, an S-Methyl Cyclohexadiene-dione from Hyalangium minutum. Journal of Natural Products 75:768–770. https://doi.org/10.1021/np200776v

Okanya PW, Mohr KI, Gerth K, Kessler W, Jansen R, Stadler M, Müller R (2014) Hyafurones, Hyapyrrolines, and Hyapyrones: Polyketides from Hyalangium minutum. Journal of Natural Products 77:1420–1429. https://doi.org/10.1021/np500145f

Pfütze S, Khamsim A, Surup F, Decock C, Matasyoh JC, Stadler M (2023) Calamene-Type Sesqui-, Mero-, and Bis-sesquiterpenoids from Cultures of Heimiomyces sp., a Basidiomycete Collected in Africa. Journal of Natural Products 86:390–397. https://doi.org/10.1021/acs.jnatprod.2c01015

Quang DN, Hashimoto T, Tanaka M, Baumgartner M, Stadler M, Asakawa Y (2002) Chemical constituents of the ascomycete Daldinia concentrica. Journal of Natural Products 65:1869–1874. https://doi.org/10.1021/np020301h

Sudarman E, Kuhnert E, Hyde KD, Sir EB, Surup F, Stadler M (2016) Truncatones A–D, benzo[j]fluoranthenes from Annulohypoxylon species (Xylariaceae, Ascomycota). Tetrahedron 72:6450–6454. https://doi.org/10.1016/j.tet.2016.08.054

Surup F, Kuhnert E, Böhm A, Pendzialek T, Solga D, Wiebach V, Engler H, Berkessel A, Stadler M, Kalesse M (2018) The Rickiols: 20-, 22-, and 24-membered Macrolides from the Ascomycete Hypoxylon rickii. Chemistry 24:2200–2213. https://doi.org/10.1002/chem.201704928

Trowitzsch W, Wray V, Gerth K, Höfle G (1982) Structure of myxovirescin A, a new macrocylic antibiotic from gliding bacteria. J. Chem. Soc., Chem. Commun.:1340–1342. https://doi.org/10.1039/C39820001340

Türck M, Bierbaum G (2012) Purification and activity testing of the full-length YycFGHI proteins of *Staphylococcus aureus*. PLoS One 7:e30403. https://doi.org/10.1371/journal.pone.0030403

Weissman KJ, Müller R (2010) Myxobacterial secondary metabolites: bioactivities and modes-of-action. Nat Prod Rep 27:1276–1295. https://doi.org/10.1039/C001260M

Xiao Y, Gerth K, Müller R, Wall D (2012) Myxobacterium-produced antibiotic TA (myxovirescin) inhibits type II signal peptidase. Antimicrob Agents Chemother 56:2014–2021. https://doi.org/10.1128/aac.06148-11

Zander W, Gerth K, Mohr KI, Kessler W, Jansen R, Müller R (2011) Roimatacene: an antibiotic against Gram-negative bacteria isolated from Cystobacter ferrugineus Cb G35 (Myxobacteria). Chemistry 17:7875–7881. https://doi.org/10.1002/chem.201003677

Zeng H, Stadler M, Abraham W-R, Müsken M, Schrey H (2023) Inhibitory Effects of the Fungal Pigment Rubiginosin C on Hyphal and Biofilm Formation in Candida albicans and Candida auris. J Fungi (Basel) 9. https://doi.org/10.3390/jof9070726
