# Supplementary material for: Construction of a novel reporter strain indicating inhibition of cell wall biosynthesis
Source: Appl Microbiol Biotechnol. 2026 Aug 28;110:260. doi: 10.1007/s00253-026-14013-7 (PMC13525142; doi:10.1007/s00253-026-14013-7)

**Figure S1: Uncropped gel image showing transphosphorylation of WalRK in the presence of selected natural products that appeared interesting *in vivo*.** The compounds were tested at the highest possible concentrations. A negative control (WalRK without ATP), a positive control (WalRK with ATP), and a DMSO control (WalRK with DMSO) were included. The gel shows dephosphorylated WalK (highest band), phosphorylated WalR (middle band), and dephosphorylated WalR (lowest band). Sulasoren A was included in the initial *in vivo* screening but showed no detectable effect under those conditions. It was nevertheless included in the *in vitro* assay for completeness, resulting in an additional lane in the uncropped gel that is not present in the cropped version shown in the Supplementary Figure S6.

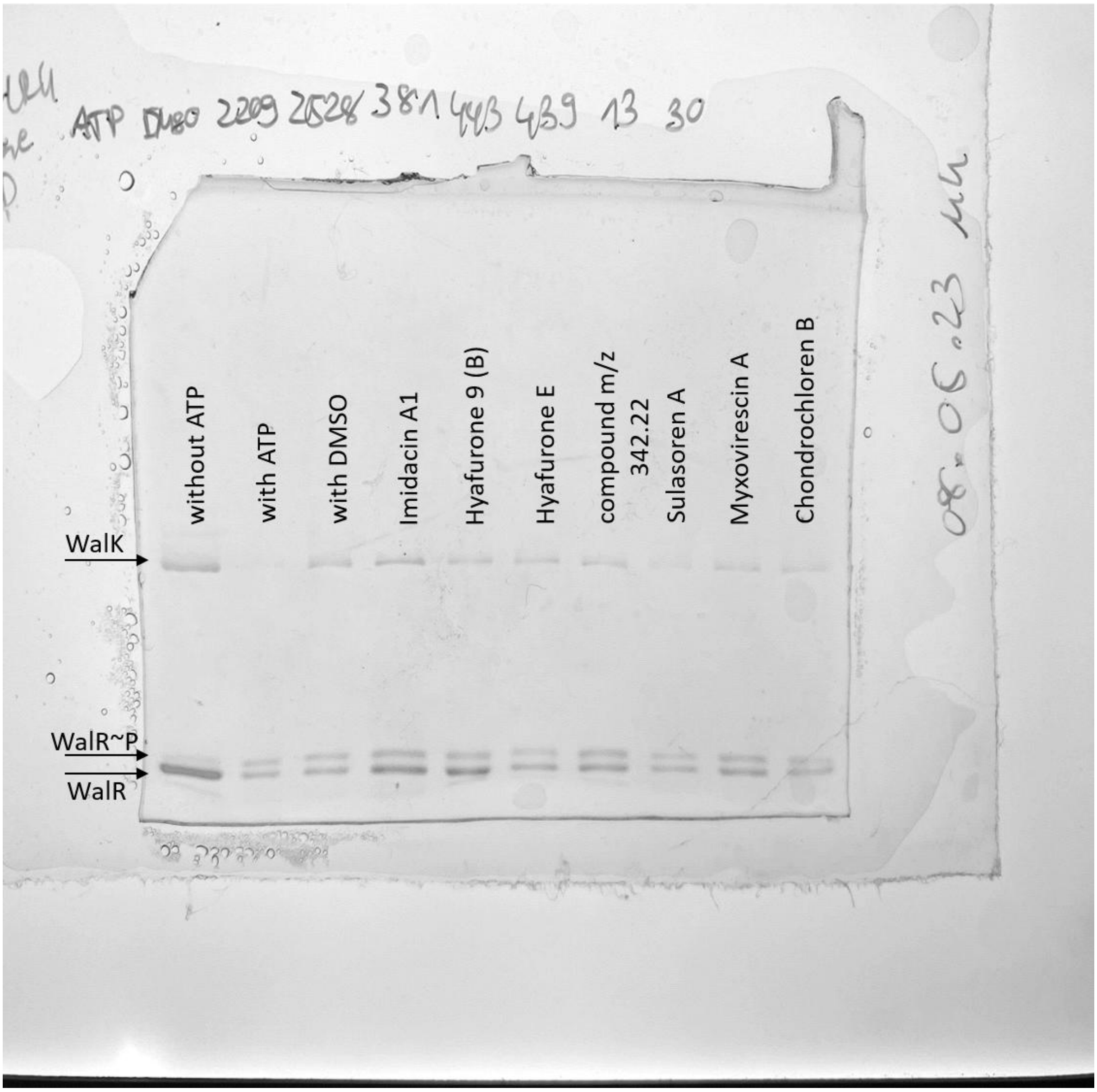

Supplement: Supplementary file 2 [file 253_2026_14013_MOESM2_ESM.pdf]
